# Matrix stiffness regulates mitochondria‐lysosome contacts to modulate the mitochondrial network, alleviate the senescence of MSCs

**DOI:** 10.1111/cpr.13746

**Published:** 2024-10-01

**Authors:** Kang Wang, Chingchun Ho, Xiangyu Li, Jianfeng Hou, Qipei Luo, Jiahong Wu, Yuxin Yang, Xinchun Zhang

**Affiliations:** ^1^ Hospital of Stomatology, Guanghua School of Stomatology Sun Yat‐sen University Guangzhou People's Republic of China; ^2^ Guangdong Provincial Key Laboratory of Stomatology Guangzhou People's Republic of China; ^3^ The Seventh Affiliated Hospital Sun Yat‐sen University Shenzhen People's Republic of China; ^4^ Department of Joint and Trauma Surgery The Third Affiliated Hospital of Sun Yat‐sen University Guangzhou People's Republic of China; ^5^ School of Medicine Sun Yat‐sen University Shenzhen People's Republic of China

## Abstract

The extracellular microenvironment encompasses the extracellular matrix, neighbouring cells, cytokines, and fluid components. Anomalies in the microenvironment can trigger aging and a decreased differentiation capacity in mesenchymal stem cells (MSCs). MSCs can perceive variations in the firmness of the extracellular matrix and respond by regulating mitochondrial function. Diminished mitochondrial function is intricately linked to cellular aging, and studies have shown that mitochondria‐lysosome contacts (M‐L contacts) can regulate mitochondrial function to sustain cellular equilibrium. Nonetheless, the influence of M‐L contacts on MSC aging under varying matrix stiffness remains unclear. In this study, utilizing single‐cell RNA sequencing and atomic force microscopy, we further demonstrate that reduced matrix stiffness in older individuals leads to MSC aging and subsequent decline in osteogenic ability. Mechanistically, augmented M‐L contacts under low matrix stiffness exacerbate MSC aging by escalating mitochondrial oxidative stress and peripheral division. Moreover, under soft matrix stiffness, cytoskeleton reorganization facilitates rapid movement of lysosomes. The M‐L contacts inhibitor ML282 ameliorates MSC aging by reinstating mitochondrial network and function. Overall, our findings confirm that MSC aging is instigated by disruption of the mitochondrial network and function induced by matrix stiffness, while also elucidating the potential mechanism by which M‐L Contact regulates mitochondrial homeostasis. Crucially, this presents promise for cellular anti‐aging strategies centred on mitochondria, particularly in the realm of stem cell therapy.

## Graphical abstract

Under soft matrix stiffness, lysosomes make more contacts with mitochondria through disorganized cytoskeleton, leading to calcium ion transfer and subsequently causing mitochondrial redox imbalance. Additionally, M‐L contact results in imbalanced mitochondrial fission dynamics, ultimately leading to the aging of MSCs.

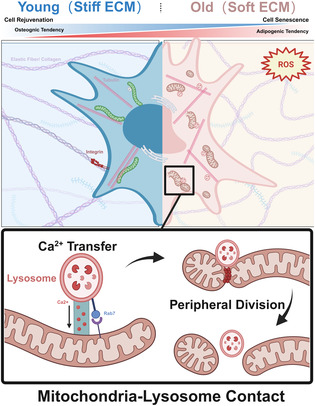

## 1 INTRODUCTION

In the realm of social development and improved infrastructure, the incidence of age‐related skeletal diseases has rapidly escalated.^1^, ^2^ Despite the cumulative progress in recent years, the microenvironmental changes in bone regeneration post‐fracture in the elderly remain a significant hindrance to achieving effective and swift recovery.^3^, ^4^

Microenvironmental alterations lead to a decrease in the osteogenic differentiation capacity of mesenchymal stem cells (MSCs), consequently compromising the MSC niche.^5^ This encompasses modifications in the extracellular matrix (ECM) and changes in the quality and quantity of collagen, elastin, and other matrix proteins, ultimately affecting the stiffness and elasticity of the ECM.^6^, ^7^ The aging process brings about an increased inflammatory response,^8^, ^9^ oxidative stress,^10^, ^11^ where excessive reactive oxygen species (ROS) can lead to imbalances in cellular redox homeostasis, affecting cell metabolism, DNA damage repair and signal transduction, and decline in stem cell function.^12^, ^13^, ^14^ It is important to emphasize that the imbalance in cellular homeostasis caused by changes in the stiffness of the extracellular matrix within the microenvironment significantly impairs the cell's anti‐aging capacity,^15^, ^16^, ^17^, ^18^ thereby reducing the osteogenic potential of MSCs.^19^, ^20^

The aberrant function of mitochondria is closely associated with the aging and differentiation tendency of MSCs.^21^ Smith et al. pointed out in their study that the decline in mitochondrial function is closely linked to the cellular aging process.^22^ Other research found that mitochondrial DNA damage and dysfunction can lead to increased intracellular oxidative stress, thereby inducing cellular aging.^23^ As individuals age, the quantity and function of mitochondria in MSCs undergo alterations, leading to an increase in intracellular ROS, thereby impacting cell function and viability.^24^ Additionally, reports indicate that reduced mitochondrial function can disrupt intracellular energy metabolism, thereby triggering cellular apoptosis and aging‐related pathways.^25^ Mitochondrial dysfunction can also lead to damage to mitochondrial DNA and variations in mitochondrial membrane permeability, ultimately affecting the function of MSCs.^26^ To systematically regulate mitochondrial function, it is crucial to downregulate the reshaping of the mitochondrial network under specific stress conditions. This process involves the cellular response to internal and external pressures, encompassing interactions between mitochondria and lysosomes, as well as reshaping the mitochondrial network at the subcellular level through mitochondrial dynamics and lysosomal dynamic movement.^27^ In fact, previous studies have largely focused on revealing the role of mitochondrial function in age‐related bone diseases, such as osteoporosis and bone tumours. Therefore, elucidating the role of mitochondrial fission in regulating mitochondrial homeostasis and MSC aging will provide new insights into the treatment of mitochondrial‐related skeletal system diseases.^28^

Recent evidence increasingly suggests that a unique non‐cellular autophagy mechanism, mitochondrial peripheral fission, characterized by lysosome‐mediated mitochondrial‐lysosomal contact, plays a pivotal role in maintaining cellular homeostasis, including in various cell types such as MSCs.^29^ Given the abundance of ROS in the aging cellular microenvironment, as well as the substantial ROS production during osteogenic differentiation, MSCs bear a hefty metabolic burden of ROS, which can be effectively managed through the formation of the mitochondrial network. These findings collectively underscore that maintaining a robust mitochondrial network may be an optimal approach to enhance cellular anti‐aging capabilities, consequently bolstering the restoration of bone regeneration capacity in elderly individuals' MSCs. Nevertheless, current research on mitochondrial network regulation has predominantly focused on scrutinizing the role or mechanisms of individual organelles in energy metabolism or the redox system through the study of isolated signalling molecules, leaving the regulatory mechanisms at the subcellular level unclear.

In this study, we scrutinized the regulatory role of ECM stiffness in maintaining mitochondrial function and observed that excessive mitochondria‐lysosome contacts increased mitochondrial fission, leading to the accumulation of ROS within the mitochondria, which mediates the aging of MSCs and reduced their tendency for osteogenic differentiation. We also proposed that cytoskeleton reorganization mediated by ECM stiffness could be a potential therapeutic strategy to regulate mitochondrial homeostasis and address MSC aging. The small molecule drug ML282 was found to alleviate the burden of mitochondrial oxidative stress and suppress mitochondrial fission, ultimately mitigating cellular aging.

Furthermore, in vivo experiments demonstrated that MSCs effectively maintained mitochondrial homeostasis, enhancing osteogenic potential following bone defects. Our findings shed light on the role of mitochondrial homeostasis in regulating MSC aging and propose a mitochondrial‐targeted therapeutic strategy based on small molecule drugs, with potential for improving MSC function and osteogenic differentiation. Future research on the molecular mechanisms regulating the relationship between mitochondria and MSC aging will contribute to providing more effective targeted strategies for the treatment of related diseases.^30^

## 2 METHODS AND EXPERIMENTS

### 2.1 Animal

C57BL/6 mice, aged 8 weeks and 24 months, were procured from Jiangsu GemPharmatech. Animals were housed in the Sun Yat‐sen University Animal Center, where they had free access to food and water, and were maintained under standard conditions of temperature (25°C), humidity (70%), and a 12‐h light/12‐h dark cycle. All procedures involving animals and their use were ethically approved by the Ethical Committee of Sun Yat‐sen University, China (Approval No. SYSU‐IACUC‐2022‐003187).

### 2.2 Cranial bone defect and MSCs transplantation

The surgical procedure for cranial bone defect was performed according to the previously established protocol.^31^ In brief, C57BL/6 mice were anaesthetised with pentobarbital sodium, and a midline sagittal incision was made on the mouse's scalp to expose the cranial bone. The 2 mm diameter cranial defect was created on the left and right parietal bones using a hand drill, taking care not to damage the underlying dura mater. The surgical site was rinsed with physiological saline, and a concentration of 10^6^/mL MSCs was implanted or no cells were implanted. The incision was tightly sutured, and penicillin injections were administered for three consecutive days postoperatively to prevent infection. The mice were closely monitored for vital signs.

### 2.3 Cell culture

In this study, Human umbilical MSCs (hUC‐MSCs) were gifted from The Biotherapy Center, the Third Affiliated Hospital, Sun Yat‐Sen University. The collected hUC‐MSCs were seeded into 75cm^2^ cell culture bottles at a density of 1 × 10^6^ cells/cm^2^. MSCs were cultured in the low‐glucose DMEM (Invitrogen) supplemented with 10% FBS (Invitrogen), 100 IU/mL penicillin (Invitrogen), and 100 μg/mL streptomycin (Invitrogen). All the cells were cultured in a humidified 5% CO_2_–95% air environment at 37°C.

For the in vitro induction of aging experiment, DMEM and hydrogen peroxide (300 μM, 40 min) were used. After drug withdrawal, the MSCs were cultured in a 5% CO_2_, 37°C incubator for 96 h before being used for the subsequent experiment.

In the context of in vitro drug treatment experiments, CID‐1067700 (HY‐13452, MCE), as a Rab7 antagonist, was utilized to reduce the generation of M‐L contacts. DMEM and CID‐1067700 (50 nM, 3 h) were employed for treating aging MSCs.

### 2.4 In vitro differentiation of hUC‐MSCs

#### 2.4.1 Osteogenic differentiation and detection

##### Osteogenic differentiation

To assess osteogenic differentiation potential, hUC‐MSCs were cultured in α‐MEM (Invitrogen) supplemented with 10% FBS (Invitrogen), 50 μg/mL ascorbic acid (Sigma), 10 mM β‐glycerophosphate (Sigma), 10 nM dexamethasone (Sigma), 100 U/mL streptomycin (Invitrogen), and 100 U/mL penicillin (Invitrogen) for a duration of 2 weeks, with regular changes of the induction medium every 3 days. Following the induction period, osteogenic‐differentiated cells were fixed, and osteoblast differentiation was assessed through alizarin red staining (ARS) and alkaline phosphatase staining (ALP). We assessed the mRNA expression levels of osteogenic‐related genes such as RUNX2, ALP, and OCN at the corresponding time points.

##### Alizarin red staining

At 28 days after the addition of osteogenic induction medium, the cells were washed three times with PBS, fixed with 4% paraformaldehyde (PFA) for 15 min, and then each well was treated with 300 μL of ARS working solution. After incubating at room temperature in the dark for 10 min, the wells were rinsed with double‐distilled water to remove excess dye and observed under a microscope for ARS staining and calcium deposition.

##### Alkaline phosphatase staining

At 14 days after the addition of osteogenic induction medium, the cells were washed three times with PBS, fixed with 4% PFA for 15 min, and then each well was treated with 300 μL of ALP staining working solution. After incubating at room temperature in the dark for 2 h, the wells were washed with PBS to remove excess dye, and observed under a microscope.

#### 2.4.2 Adipogenic differentiation and detection

To determine adipogenic differentiation potential, hUC‐MSCs were initially cultured in induction medium A comprising high‐glucose DMEM (Invitrogen), 10%FBS (Invitrogen), 0.5 mM 3‐isobutyl‐1‐methylxanthine (Sigma), 10 μg/mL insulin (Sigma), 100 nM dexamethasone (Sigma), 0.2 mM indomethacin (Sigma), 100 U/mL streptomycin (Invitrogen), and 100 U/mL penicillin (Invitrogen) for a duration of 3 days. Subsequently, cells were cultured in maintenance medium, consisting of high‐glucose DMEM (Invitrogen), 10% FBS (Invitrogen), 10 μg/mL insulin (Sigma), 100 U/mL streptomycin (Invitrogen), and 100 U/mL penicillin (Invitrogen), for 1 day, forming a cycle. This cycle was repeated thrice, followed by an additional 7 days of culture in maintenance medium alone. Adipogenic differentiation was confirmed through Oil Red O (Sigma) staining, as previously described.^32^ We assessed the mRNA expression levels of adipogenic‐related genes such as C/EBPα and PPARγ at the corresponding time points.

### 2.5 Micro CT

#### 2.5.1 In vivo

Micro‐CT was adopted to analyse bone regeneration in vivo. At postoperative days 0, 7, 14, and 28, under isoflurane gas anaesthesia, the bone recovery of the mouse cranial defect area was assessed through in vivo imaging using the Super Nova Micro‐CT system. Subsequently, bone analysis was performed using Thermo AVIZO software, and three‐dimensional reconstruction was conducted using SLICER 5.2.1 to observe bone tissue healing. Additionally, the ratio of bone volume and healed ratio of each sample was also calculated.

#### 2.5.2 In vitro

Micro‐CT was adopted to analyse bone regeneration in vivo. After 14 and 28 days post‐operation, mice were euthanized using pentobarbital sodium and cervical dislocation. The skulls of the mice were then removed and subjected to ex vivo scanning using Micro CT50 to assess bone recovery in the defect area. Subsequently, bone analysis and three‐dimensional reconstruction were performed using μCT50 software to observe the healing of the bone tissue. The ratio of bone volume/tissue volume (BV/TV), trabecular bone thickness (Tb.Th), and trabecular bone number (Tb.N) of each sample were also calculated.

### 2.6 Histological analysis

Histological analysis was performed using H&E, Masson's trichrome staining, and immunohistochemical staining for OCN. At 4 weeks postoperatively, after euthanizing the mice, the cranial bone tissues were soaked in PBS solution for washing, decalcified in EDTA solution for 21 days, fixed in 4% PFA, dehydrated, and embedded in paraffin. The paraffin‐embedded cranial bones were longitudinally sectioned into 4‐μm‐thick slices. Paraffin sections were deparaffinized in PBS and rehydrated. Haematoxylin and eosin (H&E) were used for H&E staining. Masson's trichrome staining was used to detect collagen deposition and new bone formation. OCN immunohistochemical staining was conducted to assess the osteogenic effects. The stained specimens were observed under a microscope, and the number of positive cells per area was calculated. The sections were analysed, and images were captured using Leica Aperio AT2 microscope (Leica). Subsequent image analysis was performed using ImageJ.

### 2.7 scRNA‐seq data processing

The single‐cell RNA sequencing data of mouse bone marrow MSCs was downloaded from Genome Sequence Archive in the National Genomics Data Center, China National Center for Bioinformation/Beijing Institute of Genomics, Chinese Academy of Sciences (GSA‐Human: HRA003258) with the identifier (https://ngdc.cncb.ac.cn/gsa-human/browse/HRA003258).^33^ CellRanger (version 6.1.1) was used to obtain the fastq files of the raw data and annotate them with the human genome reference sequence (GRCh38). The gene‐barcode matrix was then obtained following the Seurat (version 4.1.3) pipeline in R software (version 4.2.3). After quality control, normalization, cell clustering, and cell type annotation, we performed the following analyses: compositional analysis, differential expression genes testing, subgroup proportion calculation, functional scores analysis, and gene set enrichment. Quality control of concrete parameters is in Supplementary Table 1.

Functional scores were calculated by AUCell algorithm as the average normalized expression of the corresponding gene sets, with the full gene list provided in Supplementary Table 2. The aging‐related genes and stem cell differentiation‐related genes were collected from the online database (Gene Ontology, KEGG, and Reactome).

### 2.8 Atomic force microscopy

Three days after the injection of MSCs, mice were intravenously anaesthetised with pentobarbital sodium (35 mg/kg) and euthanized using cervical dislocation. Carefully, the mouse cranial bones were extracted and physically fixed on slides. The samples were centrifuged at 1000 rpm for 2 min to ensure cell stability at the defect site and then soaked in PBS for atomic force microscopy. The elastic modulus of the extracellular matrix at the defect site was measured using an atomic force microscope. Polystyrene microspheres with a radius of 2.5 μm were attached to a V‐shaped, tipless cantilever (MLCT‐O10) to assess the micromechanical properties of the extracellular matrix. Prior to the experiment, the probe's elastic constant was calibrated using a thermal noise method. Force curves were obtained and analysed using the built‐in software Nanoscope Analysis 2.0. The experimental setup is illustrated in Figure 2A.

### 2.9 The preparation of hydrogels with different matrix stiffness

With different ratios of acrylamide and polyacrylamide to construct two‐dimensional matrix gels with varying stiffness, as shown in the table below. After preparation, the mixture is agitated, a coagulant is added, and the resulting solution is quickly injected into a WB plate and left undisturbed for 15 min. The formed acrylamide gel is then removed and cut into pieces using a hole puncher, which are subsequently laid flat in a well plate. Following this, the gel is rinsed three times with sterile HEPES, each time for 3 min. After removing the HEPES, a UV cross‐linker (0.2 mg/mL sulfo SANPAH, dissolved in PBS) is added, and the gel is cross‐linked under UV light for at least 30 min at 10–15 cm, until the liquid changes colour from dark red to light orange, indicating successful cross‐linking. The cross‐linker is then removed, and the gel is washed twice with HEPES before adding mouse tail type I collagen (performed on ice) to cover the gel surface, followed by a 2‐h reaction at 37°C. After removing the collagen, the gel is washed three times with sterile PBS, each time for 3 min, and then stored in sterile PBS for subsequent experiments.

### 2.10 Senescence‐associated beta‐galactosidase (Sa‐β‐Gal) staining

Following the manufacturer's instructions for the senescence‐associated β‐galactosidase kit (Beyotime, #C0602), hUC‐MSCs seeded on matrigel‐coated 24‐well plates underwent two washes with PBS and fixation in stationary liquid for 15 min. Subsequently, hUC‐MSCs were exposed to a working solution of β‐galactosidase supplemented with X‐Gal and kept in a light‐protected environment at 37°C for 2 days. Senescent cells were then visualized using an optical microscope (Leika, DMi8) across five randomly selected fields of view per group, and mean values were obtained through statistical analysis.

### 2.11 RNA isolation and real‐time quantitative PCR

Total RNA was extracted from testis tissue or cells using the TRIzol reagent (Molecular Research Center, Inc.) according to the manufacturer's protocol. Quantification was performed with a NanoDrop 8000 spectrophotometer, and 1 μg of total RNA was used to reverse transcription with a RevertAid First Strand cDNA Synthesis Kit (Thermo Fisher Scientific, K1622). cDNAs were used as the template for real‐time quantitative PCR (qPCR) reactions with the FastStart Essential DNA Green Master Mix (Roche, 06924204001). All samples were run in triplicate and the results were normalized to 18 S rRNA as relative mRNA levels. The primers designed and used for qPCR are described in Supplementary Table 3.

### 2.12 Mitochondria and lysosome contact staining and analysis

The mitochondrial structural network was detected by staining with 200 nM Mitotracker Green, and lysosomes were detected by 50 nM lysotracker Red (Thermo Fisher Scientific) in a culture medium at 37 °C for 30 min. Then, the cell nuclei were stained with Hoechst 33342 (Invitrogen, 20 nM) for 15 min. Cells were imaged using a Zeiss 980 Laser Scanning Confocal Microscope with Airyscan. Super‐resolution living cell tracing images were performed using a commercial structured illumination microscope (HIS‐SIM). Acquired images for mitochondria morphology were analysed. The colocalization indexes of Pearson's and Manders' coefficients were calculated with the ImageJ colocalization analysis plugin according to previous articles. M‐L contacts were also reconstructed and analysed via surface‐surface contact site area tool by Imaris (Bitplane) following the manufacturer instructions.

### 2.13 Lysosome movement tracking and analysis

Lysosomes were stained using 50 nM Lysotracker Red (Thermo Fisher Scientific) in a culture medium at 37°C for 30 min. Lysosome movement was observed in living cells using HIS‐SIM. Additionally, time‐lapse imaging was employed to dynamically track and record lysosome behaviour within the cells. With Imaris (Bitplane) image processing and analysis software, quantitative analysis of lysosome movement trajectories, speed, direction, and other parameters was conducted, thereby providing insights into the movement patterns and behavioural characteristics of lysosomes within the cells.

### 2.14 HIS‐SIM imaging

Super‐resolution imaging was performed using commercialized HIS‐SIM, termed HIS‐SIM (High Intelligent and Sensitive SIM) provided by Guangzhou CSR Biotech Co. Ltd. Images were acquired using a 100×/1.5 NA oil immersion objective (Olympus). Cells were seeded on the gel and inverted in 8‐well chambered coverglass and maintained at 37°C and 5% CO_2_ in a humidified chamber for live SIM imaging. SIM images were collected and analysed as described previously.^34^ Sparse deconvolution was carried out to further improve the image quality.^35^

### 2.15 Transmission electron microscopy

To prepare cell samples for transmission electron microscopy, they were first fixed in 2.5% glutaraldehyde at room temperature for 2 h, followed by overnight storage at 4°C. Post‐fixation was conducted using 1% osmium tetroxide for 1 h. Subsequently, the samples underwent a meticulous washing process and dehydration through an ethanol gradient (30%, 50%, 70%, and 95%, 10 min per step). Following dehydration, the samples were embedded and polymerized at 60°C for 48 h. Ultrathin sections with a thickness of 80 nm were then carefully sliced and examined using a Tecnai 12 BioTwin transmission electron microscope (FEI Company, Eindhoven, The Netherlands) at an accelerating voltage of 120 keV.

For subsequent analysis, the morphology of the mitochondria is carefully evaluated using ImageJ software. Parameters obtained from a single field of view are averaged for further interpretation.

### 2.16 Mitochondrial calcium ions and lysosome staining and analysis

The mitochondrial calcium ions were detected by 200 nM RHOD‐2 AM and lysosomes were detected by 50 nM lysotracker green (Thermo Fisher Scientific) in a culture medium at 37°C for 30 min. Cells were imaged using a Zeiss 980 Laser Scanning Confocal Microscope with Airyscan. Acquired images for mitochondrial calcium ions were analysed.

### 2.17 Measurement of cellular ROS and mitochondrial ROS

According to the manufacturer's instructions, cellular ROS levels are determined using the CellROX assay kit (C10442, Thermofisher). In brief, cells are incubated in the dark at 37°C in DMEM containing 10 μmol/L CellROX for 30 min. After washing, labelled cells are captured by LSM980. Data are presented as mean fluorescence intensity (MFI) and analysed using ImageJ software.

Mitochondrial ROS levels are detected using the fluorescent dye MitoSOX™ Red (M36008, Invitrogen). Following the manufacturer's protocol, cells are incubated in medium containing MitoSOX™ Red for 30 min. After washing, labelled cells are evaluated using flow cytometry. Data are analysed using FlowJo v10.6.2 software.

### 2.18 Immunofluorescent staining of the cytoskeleton

Fixation of cells occurred in 4% PFA for 15 min. Subsequently, cells were incubated with fluorescent dyes according to manufacturer's instructions. For F‐actin staining, phalloidin was used. For F‐actin staining, phalloidin is used to label F‐actin filaments. As for tubulin, it is stained using primary antibody (1:1000, Cell Signalling Technology) followed by the appropriate secondary antibody Anti‐mouse IgG, HRP‐linked Antibody (1:5000, Cell Signalling Technology). Images were taken using an LSM880 confocal microscope with Airyscan (Zeiss). Live‐cell imaging was performed using a commercial SIM (HIS‐SIM) by following the previous report.^34^

### 2.19 Measurement of mitochondrial ATP

Following the manufacturer's instructions for the ATP Assay Kit (S0026, Beyotime), after centrifugation, remove the cell debris and add the supernatant to the substrate solution. Record the luminescence of the solution in an illuminometer, with each well's luminescence measured for 10 s. Use the BCA Protein Assay Kit (P0012S, Beyotime) to determine the protein content, and then convert the ATP concentration to nanomoles per milligram of protein.

### 2.20 Statistics and reproducibility

All experiments were conducted with a minimum of three biological replicates and demonstrated successful reproducibility. All data are presented as the mean ± standard deviation of at least three independent experiments. Sample sizes are indicated in the figure legends. Statistical analysis between two groups was performed using unpaired *t*‐test. For comparisons among multiple groups, one‐way analysis of variance followed by Tukey's post hoc test was employed. GraphPad software was used for data analysis. A two‐tailed *p*‐value of less than 0.05 was considered statistically significant. The level of significance defined as *p* < 0.05 (*), *p* < 0.01 (**), *p* < 0.001 (***).

## 3 RESULTS

**FIGURE 1:**
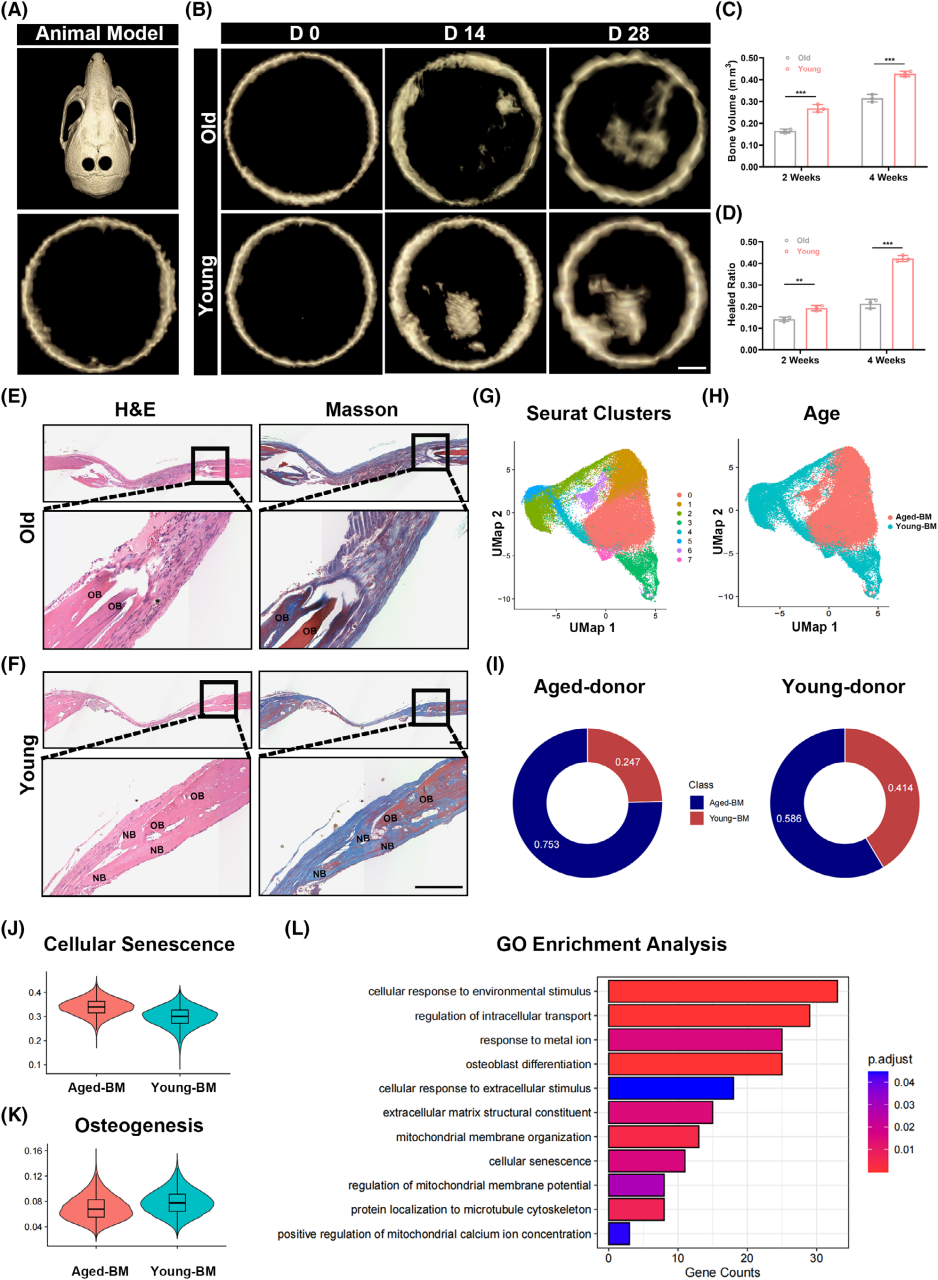
Aged MSCs contributes to the reduced osteogenic capacity observed in elderly individuals. (A) Critical‐sized calvarial defects were created in parietal bone of skeletally mature mice. (bar = 400 μm). (B) MicroCT analysis at 2 and 4 weeks revealed increased bone formation when defects were happened on younger mice. (C), (D) Analysis at 2 and 4 weeks demonstrated significantly more new bone volume deposition in the region of defect when repair happened on younger mice.(E), (F) Histological staining confirmed radiographic findings. (bar = 200 μm). (G) UMAP showing two clusters of BM‐MSCs. MSCs were displayed separately by age of samples. (H) UMAP Plot of MSCs from aged‐ samples classified based on cellular senescence status. (I) A pie chart depicting the proportions of young‐BM and aged‐BM in young and aged‐ samples. (J), (K) Violin plots of the cellular senescence score, osteogenesis score of young‐BM and aged‐BM cluster. Box plot within each violin plot indicate median values, and the 25th–75th percentiles. (L) GO enrichment analysis of the DEGs of young‐BM and aged‐BM clusters. *: *p* < 0.05, **: *p* < 0.01, ***: *p* < 0.001, ****: *p* < 0.0001, ns: no significance.

To compare the osteogenic capacity between young and aged mice, we created cranial defect models in both young and old mice without the injection of exogenous MSCs (Figure 1A). We observed that in the CT results, bone formation in young mice was significantly greater than in aged mice at 0 days, 2 weeks, and 4 weeks after surgery (Figure 1B–D). H&E and Masson staining also demonstrated more pronounced new bone formation in young mice compared to old mice (Figures 1E, F, S1C) This finding indicates that the osteogenic ability of old mice is weaker than that of young mice.

As is widely recognized, MSCs are the primary stem cells for osteogenic differentiation in vivo, and the aging status of MSCs is closely associated with osteogenesis. In order to ascertain the intrinsic reasons for the decreased osteogenic ability in aged mice, we extensively analysed the differences between young and old people BM‐MSCs by examining the single‐cell RNA sequencing data from Gao et al.^33^ (Figure S1A, B).

The gene set scoring analysis showed cells from cluster 0, cluster 1, and cluster 6 exhibit upregulated expression of cellular senescence genes (Figure S1D), so we annotated cluster 0, cluster 1, and cluster 6 as aged BM‐MSCs(aged‐BM), while the remaining clusters were labelled as young BM‐MSCs(young‐BM) (Figure 1G, H). The gene set scoring analysis showed cells from aged‐BM exhibit upregulated expression of cellular senescence genes (Figure 1J). Cell proportion analysis revealed a higher proportion of aged BM‐MSCs in samples from old donors compared to those from young donors. (Figure 1I) Differentiation‐related gene scoring analysis and gene expression bubble plots indicated lower expression of osteogenic and chondrogenic genes and higher expression of adipogenic genes in aged BM compared to young BM (Figures 1K and S1E–G). This suggests that the decline in osteogenic capacity in old individuals may be related to the aging of endogenous stem cells.

**FIGURE 2:**
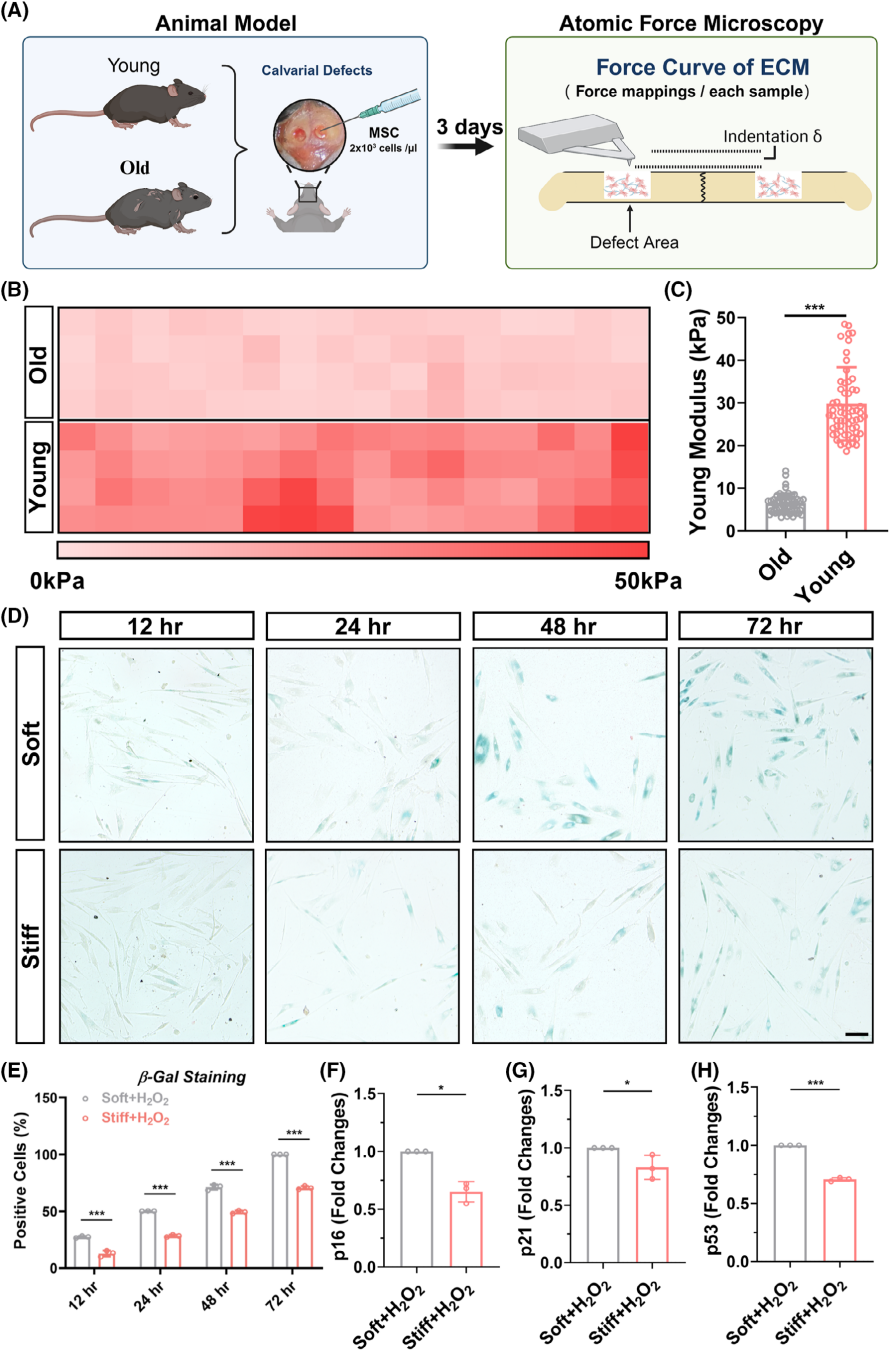
The aging of MSCs is due to the softness of the extracellular matrix in elderly individuals. (A) After injecting exogenous stem cells to repair cranial defects in young and aged mice, an illustrative diagram of atomic force microscopy measurement of extracellular matrix stiffness was created 3 days later. (B) Thermal map of the matrix stiffness of the extracellular matrix (ECM) at the site of mesenchymal stem cells (MSCs) in the cranial defect model of young and aged mice. (C) Quantitative statistical graph of figure (B). (D) The β‐gal staining images of MSCs on soft and stiff polyacrylamide gels induced by H_2_O_2_ at 12, 24, 48, and 72 h. (bar = 100 μm). (E) The proportion of β‐gal‐positive cells of MSCs on soft and stiff polyacrylamide gels induced by H_2_O_2_ at 12, 24, 48, and 72 h. (F), (H**)** qPCR analysis of relative mRNA expression of cellular senescence‐related genes, including P16, P21, P53, in MSCs on soft and stiff polyacrylamide gels induced by H_2_O_2_ at 72 h. *: *p* < 0.05, **: *p* < 0.01, ***: *p* < 0.001, ****: *p* < 0.0001, ns: no significance.

To further investigate the intrinsic causes of aging in endogenous stem cells, we conducted additional GO enrichment analysis on differentially expressed genes in aged‐BM and young‐BM. The enrichment analysis converged on cellular responses to environmental stimuli, responses to extracellular stimuli, extracellular matrix organization, and mitochondrial‐related terms, indicating a potential association between MSC aging and the extracellular microenvironment (Figure 1L). As is well known, a high matrix stiffness in the extracellular environment can promote osteogenesis in MSCs.^36^, ^37^ To confirm whether changes in stiffness contribute to the aging process during MSC osteogenesis, we treated young and aged mice with cranial defects using exogenously injected MSCs and assessed the matrix stiffness of the MSCs' extracellular matrix at the defect site using atomic force microscopy (Figure 2A). The results showed that the matrix stiffness of the extracellular matrix of MSCs at the cranial defect site in young mice (approximately 30 kPa) was greater than that in old mice (approximately 5 kPa) (Figure 2B, C). Consequently, we separately cultured MSCs on polyacrylamide gels with stiffness values of 5 and 30 kPa and induced aging using hydrogen peroxide. β‐galactosidase staining demonstrated a higher number of positively stained cells in MSCs cultured on soft polyacrylamide gels after induced aging (Figure 2D, E). Furthermore, qPCR analysis of aging‐related genes (P16, P21, and P53) showed higher expression levels in MSCs cultured on soft polyacrylamide gels (Figure 2F–H). This suggests that the aging process during MSC osteogenesis may be attributed to the softness of the extracellular matrix stiffness.

**FIGURE 3:**
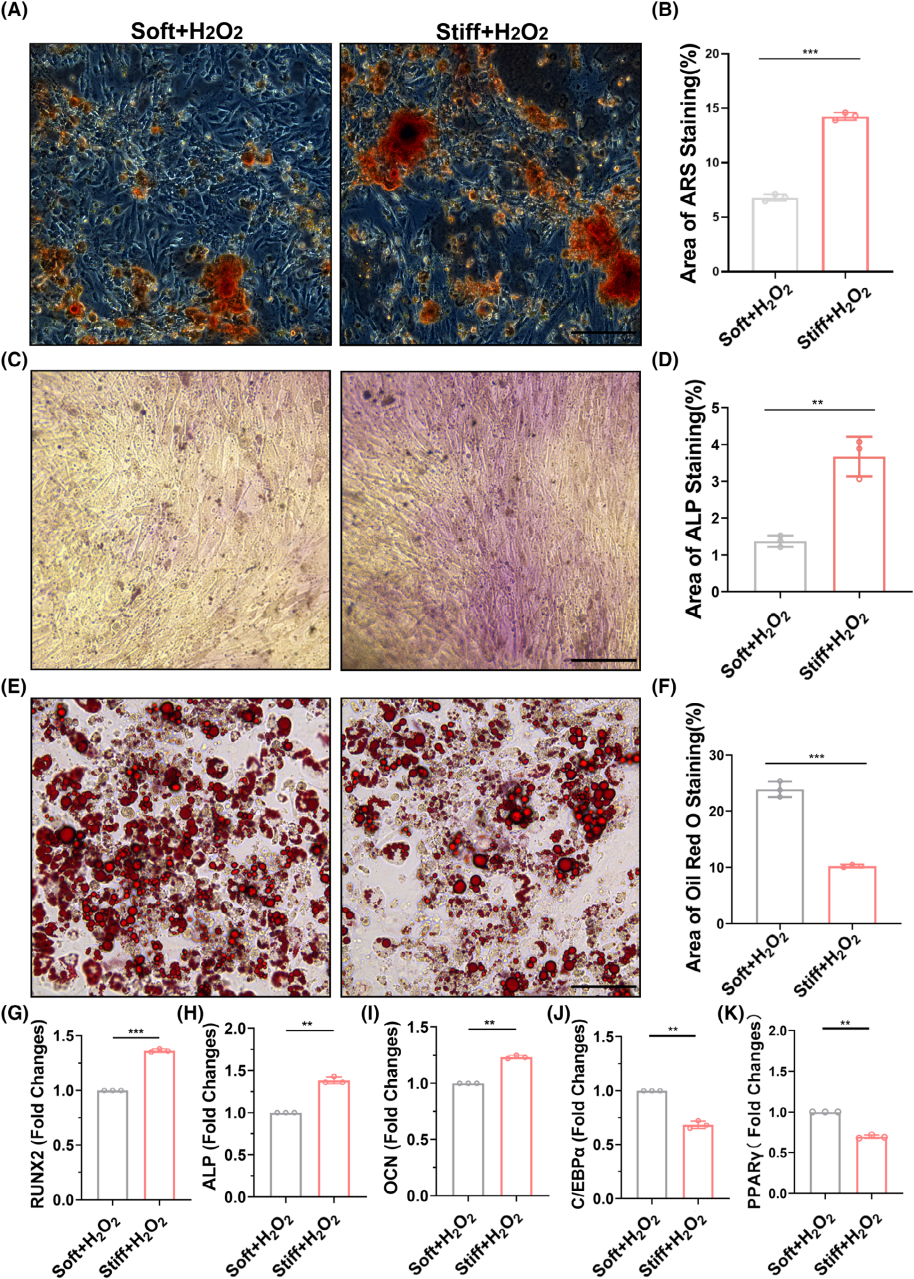
Differentiation tendencies of MSCs on soft and hard matrix stiffness. (A), (B) Representative micrograph of Alizarin red staining (red) and quantitative analysis of osteogenic differentiation induced in MSCs on soft and stiff polyacrylamide gels after H_2_O_2_ induction. (C), (D) Representative micrograph of ALP staining (purple) and quantitative analysis of osteogenic differentiation induced in MSCs on soft and stiff polyacrylamide gels after H_2_O_2_ induction. (bar = 200 μm). (E), (F) Representative micrograph of oil red staining (red) and quantitative analysis of osteogenic differentiation induced in MSCs on soft and stiff polyacrylamide gels after H_2_O_2_ induction. (bar = 200 μm). (G), (K**)** qPCR analysis of relative mRNA expression of osteocytes‐related genes (Runx2.ALP, OCN) AND adipocytes related genes(PPARγ, C/EBPα) in MSCs differentiation on soft and stiff polyacrylamide gels after H_2_O_2_ induction at 72 h. (bar = 200 μm). *: *p* < 0.05, **: *p* < 0.01, ***: *p* < 0.001, ****: *p* < 0.0001, ns: no significance.

Furthermore, we confirmed the difference in the differentiation tendency of MSCs in vitro by culturing them on soft and hard polyacrylamide gels. Both ARS and alkaline phosphatase (ALP) staining demonstrated that hydrogen peroxide‐induced MSCs cultured on hard polyacrylamide gels exhibited more calcium deposition (Figure 3A, B) and higher ALP activity compared to those cultured on soft polyacrylamide gels (Figure 3C, D). Oil Red O staining revealed that hydrogen peroxide‐induced MSCs cultured on hard polyacrylamide gels had fewer lipid droplets than those cultured on soft polyacrylamide gels (Figure 3E, F). QPCR analysis indicated that the expression of osteogenic genes in hydrogen peroxide‐induced MSCs cultured on hard polyacrylamide gels was higher than that in MSCs cultured on soft polyacrylamide gels (Figure 3G–I), whereas the expression of adipogenic genes showed the opposite trend (Figure 3J, K). This suggests that a stiffer matrix stiffness endows MSCs with greater anti‐aging capability, thereby promoting osteogenic differentiation.

**FIGURE 4:**
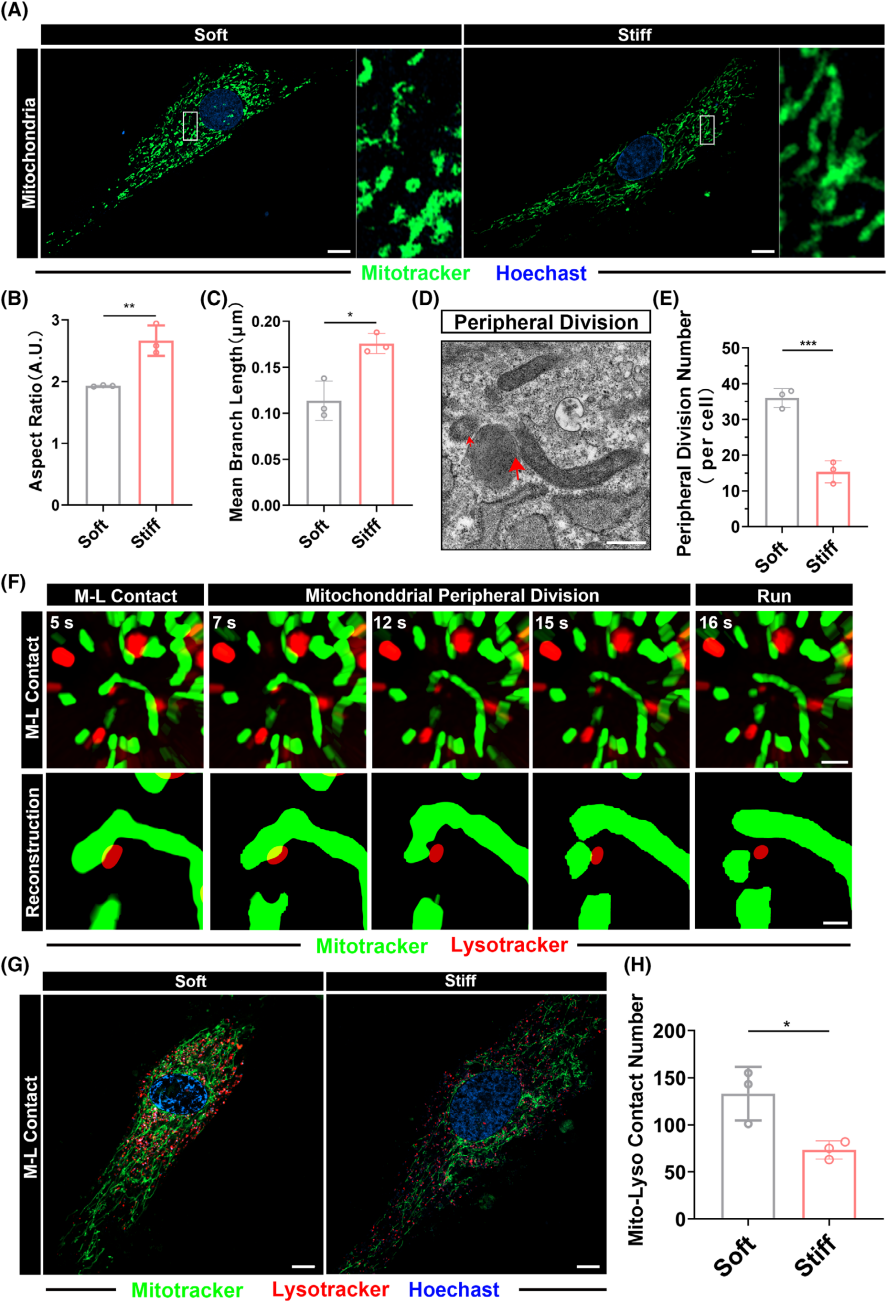
The mitochondria‐lysosome contact regulates the mitochondrial network. (A) Representative confocal microscopy images of MSC mitochondria (Mitotracker green+) on soft and stiff polyacrylamide gels induced by H_2_O_2_ at 48 h. Scale bar = 12 μm for original pictures and 1 μm for enlarged pictures. (B), (C) Quantitative statistical analysis of mitochondrial morphology in figure (A). (D) Representative TEM images of the mitochondrial peripheral division of MSCs on polyacrylamide gels induced by H_2_O_2_ at 48 h. Red arrows indicate contact area, respectively. Scale bar, 500 nm. (E) Quantitative statistical graph of mitochondrial peripheral division numbers in figure (D). (F) Representative super‐resolution living cell tracing images and reconstruction images of mitochondrial peripheral division in MSCs on polyacrylamide gels induced by H_2_O_2_ at 48 h at different time points. Mitochondria were stained with Mitotracker green and lysosome were stained with Lysotracker red. Scale bar = 1 μm for original pictures and 0.4 μm for enlarged pictures. (G) Representative confocal microscopy images of MSC M‐L contact on soft and stiff polyacrylamide gels induced by H_2_O_2_ at 48 h. Scale bar, 20 μm. (H) Quantitative statistical chart of M‐L contact numbers in figure (G). *: *p* < 0.05, **: *p* < 0.01, ***: *p* < 0.001, ****: *p* < 0.0001, ns: no significance.

Gene enrichment analysis revealed a close association between the aging of MSCs and mitochondrial‐related terms (Figure 1L). Numerous studies have indicated that the aging process is accompanied by changes in mitochondrial function and morphology. We observed the mitochondrial morphology of hydrogen peroxide‐induced MSCs cultured on soft and stiff polyacrylamide gels using high‐resolution microscopy (mitotracker green staining for mitochondria, hoechst staining for cell nuclei). The experiment results indicate that MSCs cultured on stiff polyacrylamide gels exhibit a more regular mitochondrial morphology during osteogenic differentiation, with an increase in fusion capacity, average mitochondrial length, aspect ratio, and the formation of mitochondrial network (Figure 4A–C). This conclusion has been confirmed by quantitative analysis (Figure S2A, B).

Recent research has demonstrated that physical interaction between lysosomes and mitochondria can regulate the morphology and function of the mitochondria.^27^, ^29^ In a study by Zou et al.,^38^ it was found that under pathological conditions, contact with lysosomes leads to the outer membrane fission of mitochondria, promoting both peripheral division through mitochondria‐lysosome contacts. We aimed to investigate whether this phenomenon also occurs in the context of matrix stiffness‐mediated mitochondrial homeostasis. Our observations through electron microscopy showed peripheral division at M‐L contact sites (Figure 4D), alongside central division (Figure S2A). Further statistical analysis indicated a higher occurrence of peripheral division on softer polyacrylamide gels (Figure 4E). Subsequently, we conducted delayed imaging of mitochondrial and lysosomal contact in hydrogen peroxide‐induced MSCs cultured on both soft and stiff polyacrylamide gels (Figure 4F), as well as a three‐dimensional reconstruction of mitochondrial‐lysosomal contacts (Figure S2B) to confirm the existence of M‐L contacts. Additionally, we found that this type of contact is unrelated to mitochondrial autophagy, as there was no lysosomal engulfment of mitochondria, and the two organelles separated shortly after contact (Figure S2C). Importantly, this mode of contact may lead to non‐degradative pathways or mitochondrial division. To compare the difference in the number of contacts mediated by stiffness, we conducted microscopic imaging of mitochondria and lysosomes in hydrogen peroxide‐induced MSCs cultured on both soft and stiff polyacrylamide gels (mitochondria stained with Mitotracker Green, lysosomes stained with Lysotracker Red, and cell nuclei stained with Hoechst) (Figure 4G, H). The statistical results indicated that softer polyacrylamide gels led to a higher number of mitochondria‐lysosome contacts in MSCs. This suggests that mitochondrial dysfunction induced by M‐L contacts on soft polyacrylamide gels may contribute to aging.

**FIGURE 5:**
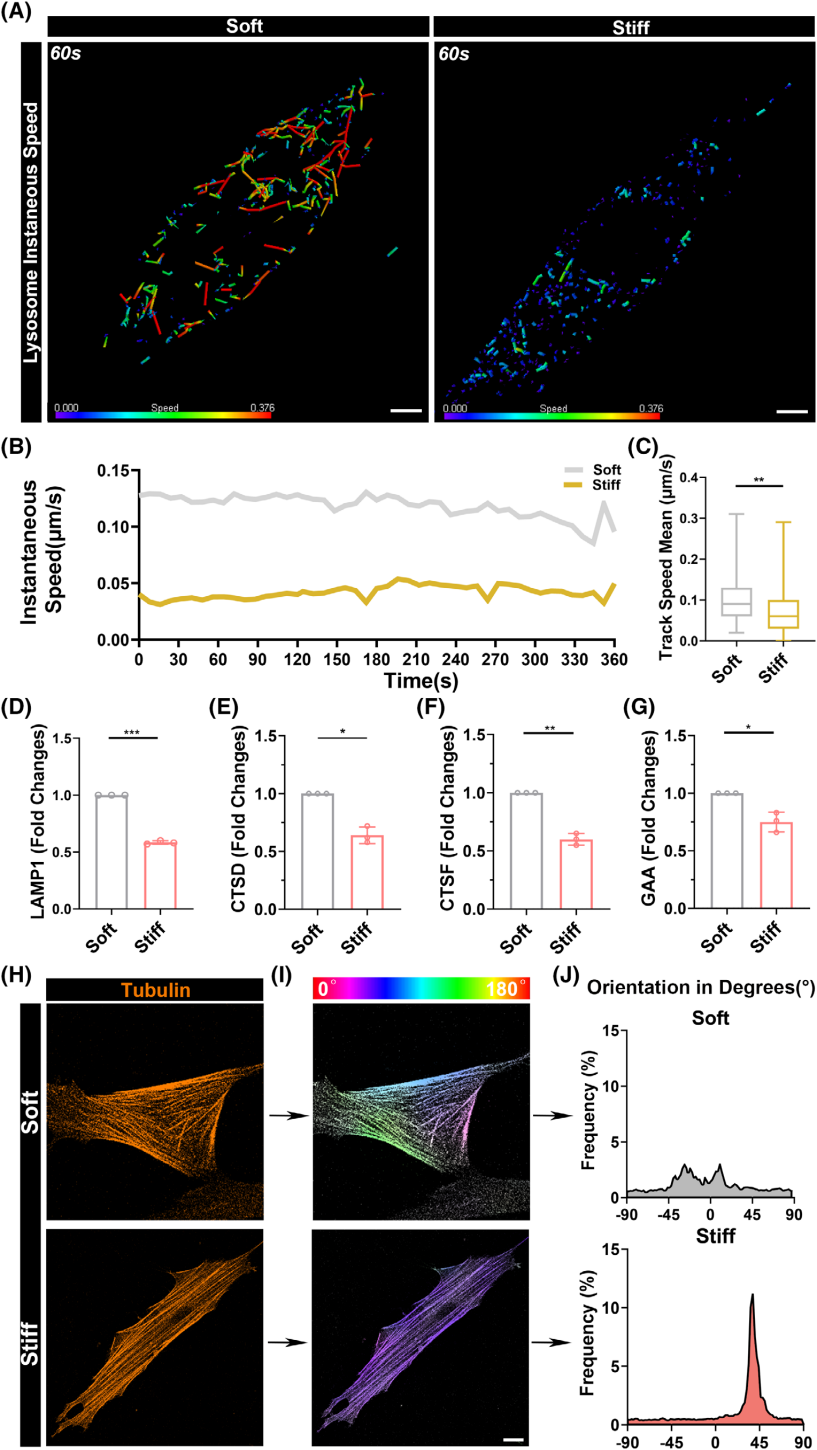
The cytoskeleton mediates lysosomal dynamics. (A) The representative super‐resolution living cell tracing image at the 60th second after 48 h of H_2_O_2_ induction shows the instantaneous velocity of lysosome movement. Lysosomes were stained with Lysotracker Red. The colour scale represents the instantaneous velocity of the current lysosomes, and the length represents the movement distance and position at the next time point. The scale bar for the original image is 10 μm. (B) Quantitative statistical graph of the instantaneous speed at each time point of figure (A). (C) Quantitative statistical graph of the track speed mean of figure (A). (D)–(G) qPCR analysis of relative mRNA expression of lysosome genes, including LAMP1, CTSD, TSF, GAA in MSCs on soft and stiff polyacrylamide gels induced by H_2_O_2_ at 72 h. (H) MSCs stained for tubulin on soft and stiff substrates. The scale bar for the original image is 10 μm. (I) Corresponding orientation plots for tubulin staining, where the different colours indicate different orientations of tubulin as per the given colourmap. Tubulin stress fibre microdomains can be identified by the uniformly coloured zones in the orientation plots. (J) Quantitative statistical graph for tubulin staining orientations of figure (I). *: *p* < 0.05, **: *p* < 0.01, ***: *p* < 0.001, ****: *p* < 0.0001, ns: no significance.

To further explore the specific reasons for the difference in the quantity of mitochondria‐lysosome, we tracked the movement rates of individual lysosomes under the regulation of soft and hard substrates using HIS‐SIM. According to the analysis of instantaneous velocity at the 60th second of time‐lapse imaging, the lysosomes on the soft polyacrylamide gel exhibited significantly higher instantaneous velocity compared to those on the rigid substrate (Figure 5A). Further analyses of instantaneous velocity at various time points and average velocity also supported this result (Figure 5B, C). Subsequently, we observed and analysed the movement distance of lysosomes on the soft polyacrylamide gel and found that the lysosomes on the soft substrate not only had higher velocity, but also moved a greater distance compared to those on the hard substrate (Figure S3A, C). We speculate that such high velocity and movement distance are due to the increased activity of lysosomes stimulated by the microenvironment. However, the reduced sensitivity of cells to ROS under high rigidity led to a decrease in the expression of lysosome‐related genes (Figure 5D–G). The heightened activity of lysosomes increased the contact with mitochondria, enhancing the opportunities for contact. Additionally, the larger cellular capacity resulting from high‐speed movement led to further imbalance in cellular homeostasis due to the increased production of ROS. However, it is evident from the movement trajectory map that the movement of highly active lysosomes is more disorderly and less organized compared to the movement of lysosomes under high rigidity (Figure S3A). Therefore, we hope to further understand how lysosomes sense and move in response to the rigidity regulation of the microenvironment. Literature indicates that lysosomes can be transported by the cytoskeleton, which can also sense changes in the rigidity of the surrounding microenvironment and rearrange accordingly.^39^, ^40^, ^41^ Thus, through immunofluorescence techniques, we discovered that the cytoskeleton on the soft substrate is more disordered, whereas on the hard substrate, the cytoskeleton is more orderly arranged (Figure 5H). Statistical results also support the disordered arrangement of the cytoskeleton on the soft polyacrylamide gel (Figures 5I, J and S3D, E). This may hinder the systematic metabolism and contact with mitochondria by the rapidly moving lysosomes, leading to the imbalance in cellular homeostasis by failing to metabolize excessive ROS.

**FIGURE 6:**
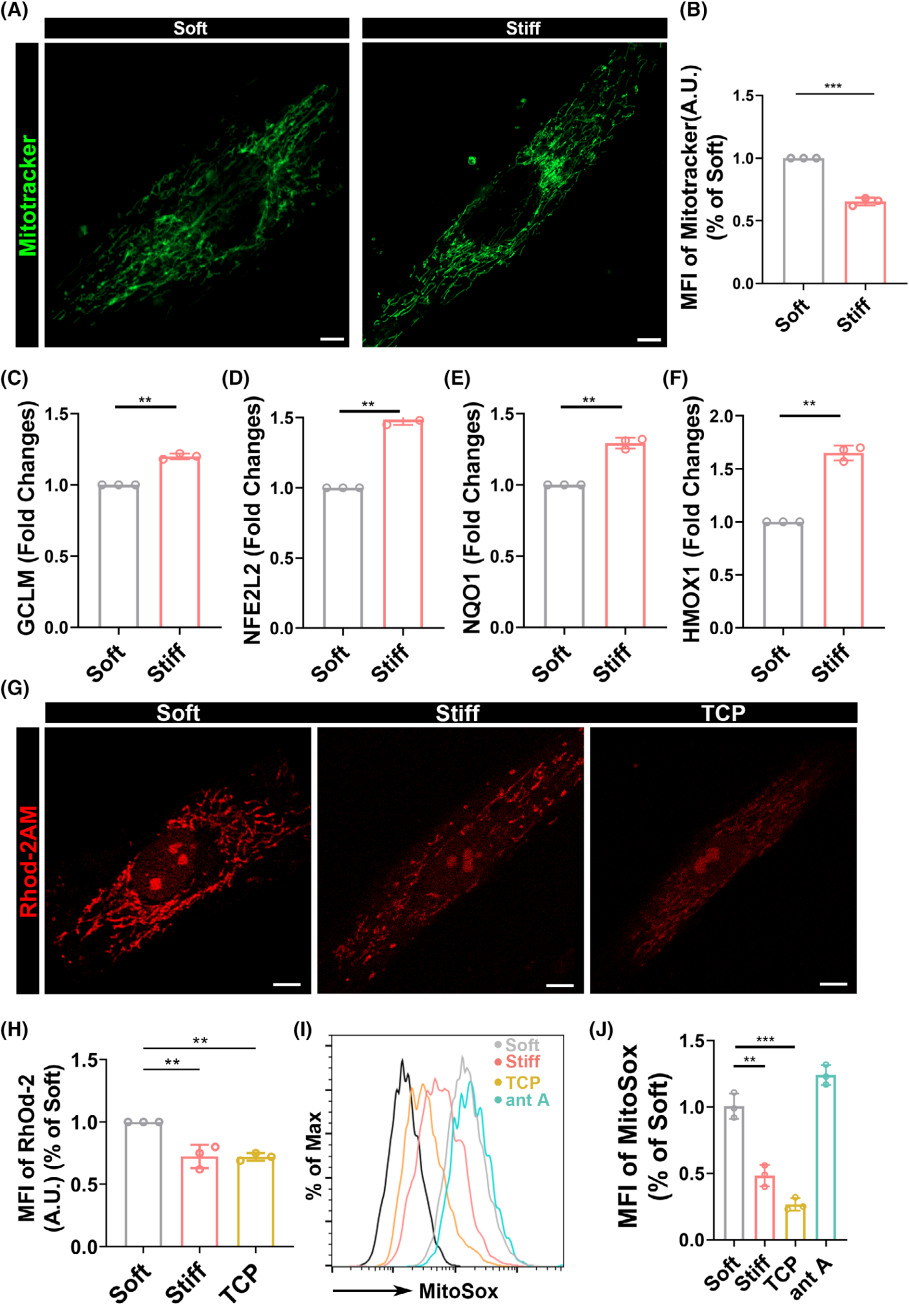
The morphology and function of mitochondria under different substrate stiffness. (A) Representative confocal microscopy images of MSCs mitochondrial morphology (Mitotracker green+) on soft and stiff polyacrylamide gels induced by H_2_O_2_ at 48 h. Scale bar, 500 nm. (B) Quantitative statistical analysis of median fluorescence intensity in figure (A). (C)–(F) qPCR analysis of relative mRNA expression of mitochondrial antioxidant enes (NFE2L2, GCLM, HMOX1, and NQO1) in MSCs on polyacrylamide gels induced by H_2_O_2_ at 48 h. (G) Representative confocal microscopy images of MSCs calcium ion (Rhod‐2AM, red+) on soft and stiff polyacrylamide gels induced by H_2_O_2_ at 48 h. Scale bar. (H) Quantitative statistical analysis of median fluorescence intensity in figure (G). TCP:tissue culture plate. (I), (J) Flow cytometry and quantitative analysis of the mean fluorescence intensity of mitochondrial ROS level in MSCs on polyacrylamide gels induced by H_2_O_2_ at 48 h. Mitochondrial ROS was probed with Mitosox*: *p* < 0.05, **: *p* < 0.01, ***: *p* < 0.001, ****: *p* < 0.0001, ns: no significance. ant A: antimycin A.

Aged cells tend to have larger mitochondrial mass, which is consistent with our experimental results,^42^ where the mitochondrial mass of MSCs on soft polyacrylamide gel was greater than that on the hard substrate (Figure 6A, B). Furthermore, the aged environment is characterized by a high level of ROS, and during the osteogenic differentiation of MSCs, oxidative phosphorylation is the primary mode of energy metabolism, leading to a substantial production of ROS. As the primary regulator of cellular redox capacity, mitochondria's production of reducing agents can reflect their redox capacity. The modifier subunit of glutamate‐cysteine ligase (GCLM) and the catalytic subunit of glutamate‐cysteine ligase (GCLC) together form the glutamate‐cysteine ligase, a key rate‐limiting enzyme in the synthesis of glutathione (GSH). Upregulation of GCLM can ameliorate iron‐induced cell death under ROS stress. The results indicate that the expression levels of genes related to redox capacity, namely GCLM, NFE2L, NQO1, and HMOX1, were higher on the hard polyacrylamide gel (Figure 6C–F). In the face of hydrogen peroxide‐induced oxidative stress, the regulation of mitochondrial redox homeostasis was found to be poorer. Subsequently, we conducted flow cytometry to analyse the ROS content in the mitochondria and found that the mitochondria of MSCs on the hard polyacrylamide gel accumulated more ROS (Figure 6I, J). Further intracellular ROS staining using CELLROX also suggested higher intracellular ROS levels on the soft polyacrylamide gel. We speculate that this may be due to the weakened redox capacity on the soft polyacrylamide gel (Figure S4C, D). Literature indicates that the calcium ion homeostasis within mitochondria is a factor associated with the ROS signalling pathway.^43^ Mitochondrial calcium overload can lead to mitochondrial dysfunction and cellular aging. Therefore, we further examined the calcium ion content within the mitochondria using the RHOD‐2AM dye. The results showed that the mitochondrial calcium ion content on the soft polyacrylamide gel was significantly higher, approaching the fluorescence expression level of the positive control (Figure 6G, H). Corresponding to mitochondrial calcium, the ATP production in mitochondria of MSCs on soft polyacrylamide gels is significantly lower than that of cells cultured on hard polyacrylamide gels (Figure S4E). Interestingly, we sought to understand the role of mitochondrial‐lysosomal contact and found that the contact between mitochondria and lysosomes may be one of the reasons for the imbalance in mitochondrial calcium ion homeostasis. We observed that the fluorescence intensity of mitochondrial calcium ions associated with mitochondrial‐lysosomal contact increased over time, whereas the increase was limited in the non‐contact group (Figure S4A, B).

**FIGURE 7:**
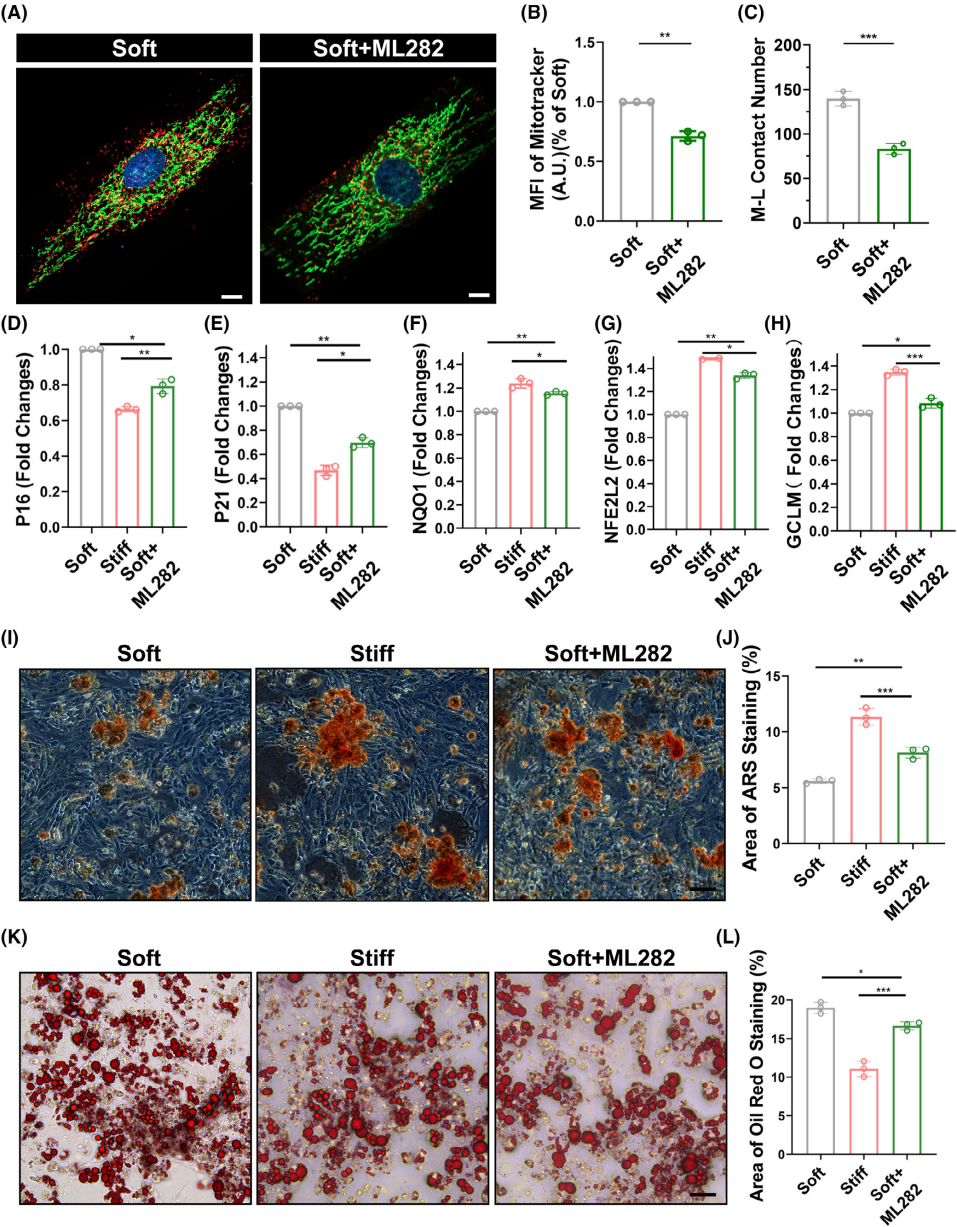
ML282 mitigates senescence in MSCs by inhibiting mitochondrial‐lysosomal contact. (A)–(C) Representative confocal microscopy images and quantitative analysis of MSC M‐L contact (Mitotracker green+, Lysotracker red+) on soft and soft+ML282 polyacrylamide gels induced by H_2_O_2_ at 48 h. (D)–(H) qPCR analysis of relative mRNA expression of cellular senescence‐related genes (P16 and P21) and mitochondrial antioxidant genes (NFE2L2, GCLM, and NQO1) in MSCs on soft and soft+ ML282 polyacrylamide gels induced by H_2_O_2_ at 72 h (*n* = 3 biological repeats for each group; ordinary one‐way ANOVA). (I), (J) Representative micrograph of Alizarin red staining (red) and quantitative analysis of osteogenic differentiation induced in MSCs on soft and soft+ ML282 polyacrylamide gels after H_2_O_2_ induction. (K), (L) Representative micrograph of oil red staining (red) and quantitative analysis of osteogenic differentiation induced in MSCs on soft and soft+ ML282 polyacrylamide gels after H_2_O_2_ induction. (*p* < 0.05, **: *p* < 0.01, ***: *p* < 0.001, ****: *p* < 0.0001, ns: no significance.)

The previous experiments have demonstrated that the peripheral division of mitochondria is caused by the contact between mitochondria and lysosomes, and the high concentration of calcium ions within the mitochondria is due to calcium ion transfer. Therefore, we targeted the contact site between mitochondria and lysosomes, a molecule presents on the lysosome, Rab7, and competitively inhibited this site using a small molecule drug (ML‐282). Firstly, through immunofluorescence techniques, we observed changes in the contact between the mitochondrial network and the mitochondrial‐lysosomal contact under the regulation of the small molecule drug, as observed through live cell staining with MitoTracker and LysoTracker (Figure 7A). Further statistical analysis revealed a reduction in the number of mitochondrial‐lysosomal contacts and a decrease in fluorescence intensity (Figure 7C). The morphology of the mitochondrial network became elongated, and the number of punctate mitochondria also decreased (Figure 7A, B). Subsequently, we further investigated whether mitochondrial redox capacity had improved and found that MSCs pretreated with ML282 exhibited an appropriate enhancement in mitochondrial redox capacity (Figure 7F–H). Additionally, through retrograde detection of two aging‐related genes, p16 and P21, we observed a decrease in their expression levels (Figure 7D, E), indicating that the improved mitochondrial network could to some extent resist ROS in the microenvironment. Finally, we examined the cell differentiation tendency. First, staining with Alizarin Red revealed that MSCs pretreated with ML282 exhibited a higher osteogenic differentiation capability and more calcium deposition compared to MSCs cultured on the soft polyacrylamide gel, although it did not reach the level of those on the hard polyacrylamide gel (Figure 7I, J). Oil Red O staining results indicated a decrease in the adipogenic potential of MSCs after pretreatment. Statistical results showed a decrease in adipogenic capability after ML282 pretreatment, although it remained higher than that on the hard polyacrylamide gel (Figure 7K, L). We speculate that this may be due to the inherent differences in substrate rigidity.

**FIGURE 8:**
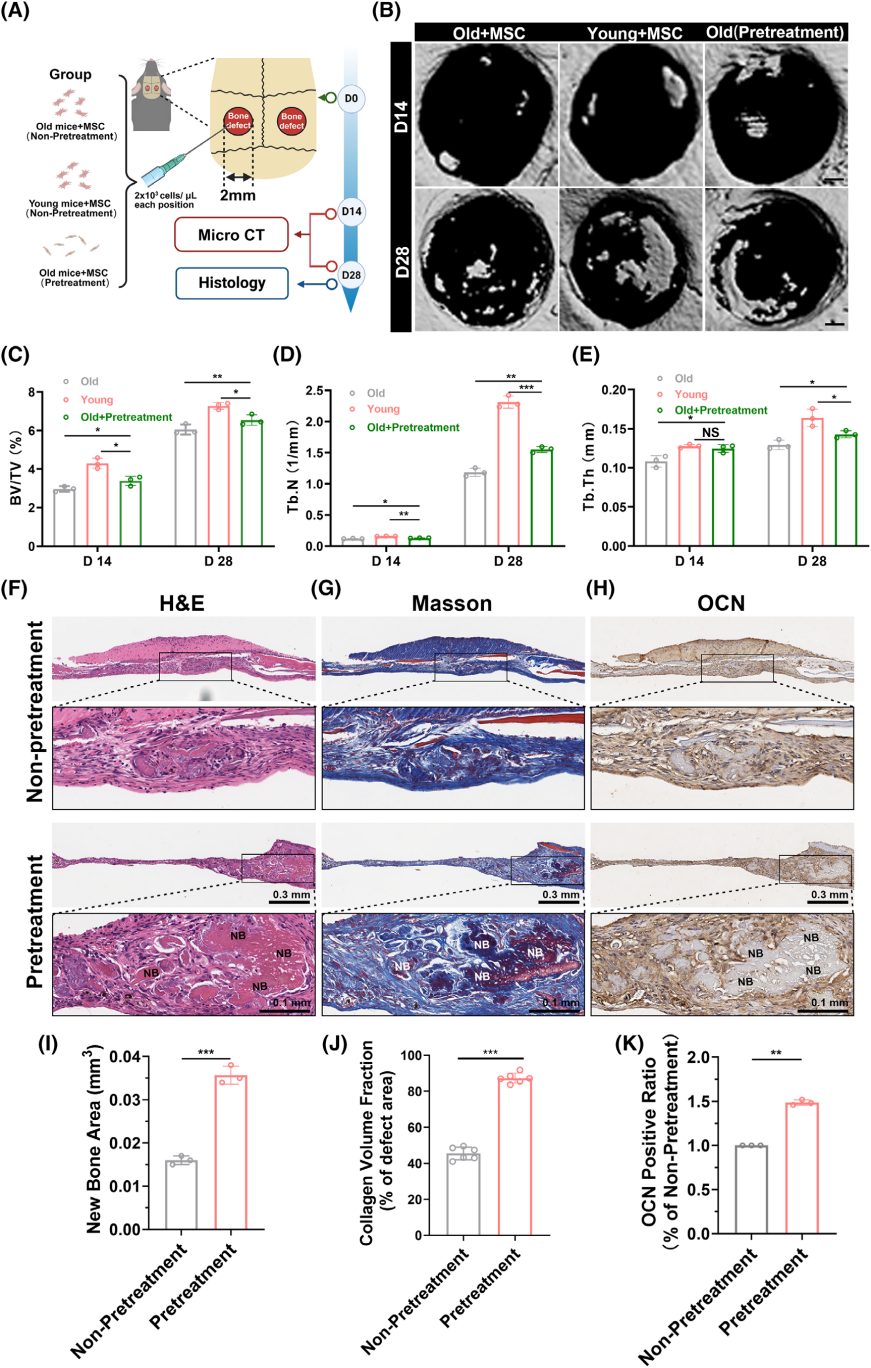
Preconditioning of MSCs with ML282 restores their osteogenic capacity in aged mice. (A) The schematic diagram of in vitro pre‐treated MSCs for the treatment of cranial defects in mice. (B) The CT reconstruction images of cranial defects in young and old mice treated with in vitro pre‐treated MSCs at D14 and D28. (C)–(E) Quantitative analysis of bone volume fraction (BV/TV), trabecular number (Tb.N) and trabecular thickness (Tb.Th) in figure (B). (F)–(K) Histological and quantitative analysis images of the cranial defects in the pre‐treatment and non‐pretreatment groups using haematoxylin and eosin (H&E), Masson, and OCN tissue staining. Scale bar, 0.3 mm for original pictures and 0.1 mm for enlarged pictures. (*p* < 0.05, **: *p* < 0.01, ***: *p* < 0.001, ****: *p* < 0.0001, NS: no significance.)

To further investigate whether reducing the contact between mitochondria and lysosomes in MSCs can resist the aging osteogenic microenvironment, we divided the experiment into three groups: young mice injected with MSCs, old mice injected with MSCs, and old mice injected with MSCs pretreated with ML282 for 2 h (Figure 8A). CT results showed that on the 28th day after skull defect, young mice had the most significant bone formation, and although the osteogenic capacity of old mice was poor, some new bone formation was still observed, indicating a certain therapeutic effect of MSC injection itself (Figure 8B–E). Additionally, for the group that underwent drug pretreatment, we found that their osteogenic capacity was superior to the old age group, although it did not reach the osteogenic capacity of young mice, it still partially restored its osteogenic efficacy (Figure 8B–E). We speculate that this may be because MSCs pretreated with the drug can better resist ROS in the aging microenvironment, although the extracellular matrix stiffness in the microenvironment remains low, resulting in weaker osteogenic differentiation capability of MSCs compared to the young group. Histological staining with HE and Masson's trichrome further confirmed that young mice exhibited more pronounced new bone formation compared to old mice, and the OCN group showed an increase in the surrounding positive cells during new bone formation (Figure 8F–K).

## 4 DISCUSSION

The mitochondrion plays a crucial role in the function and maintenance of MSCs. Research has shown that mitochondrial dysfunction is closely associated with the aging and decreased osteogenic differentiation capacity of MSCs.^23^ As MSCs age, the quantity and function of their mitochondria undergo changes, leading to an increase in ROS levels within the cells, which can affect cellular function and viability.^24^ The increase in intracellular ROS in MSCs has been found to hinder osteogenesis while promoting adipogenesis. The formation of the mitochondrial network is regulated through mitochondrial dynamics and is closely linked to the maintenance of mitochondrial homeostasis.^44^ Studies have indicated that alterations in the mitochondrial network can lead to a decrease in ATP synthesis capacity within the cell, impacting the energy supply required for osteogenic differentiation.^45^, ^46^ Moreover, mitochondrial dysfunction affects the osteogenic differentiation of MSCs, thereby influencing the regenerative and reparative capabilities of bone tissue.^47^ Osteogenic differentiation is an energy‐demanding process, and mitochondria, as the cellular energy production centre, shift from primarily relying on glycolysis to predominantly utilizing aerobic phosphorylation for energy metabolism during osteogenesis, resulting in the generation of a large amount of ROS in this process.^48^ We have confirmed that during osteogenesis, modulating the morphology of the mitochondrial network through small molecule drugs can assist cells in resisting ROS in the microenvironment and the generation of ROS during differentiation, thereby maintaining the homeostasis of MSCs and reducing susceptibility to aging. Sustaining cellular homeostasis will enable MSCs to maintain their osteogenic differentiation potential under osteogenic conditions.

The functions of mitochondria and lysosomes are closely related and vital for maintaining cellular homeostasis.^49^, ^50^ Previous studies have shown that mitochondria and lysosomes can interact directly through degradative pathways such as mitophagy or mitochondrial‐derived vesicles (MDVs), as well as through non‐degradative pathways, including mitochondria‐lysosome contact.^27^, ^28^, ^51^, ^52^ While degradative pathways have been extensively studied in cellular aging, the role of non‐degradative pathways remains less understood.^53^, ^54^ While degradative pathways have been extensively studied in cellular aging, the role of non‐degradative pathways remains less understood.^55^, ^56^ It has been reported that mitochondrial‐lysosomal contacts can facilitate the transfer of calcium ions and amino acids, and have been shown to mark the sites of mitochondrial peripheral division.^29^, ^57^, ^58^ Our work has demonstrated an increase in mitochondrial‐lysosomal contacts under soft matrix‐induced aging, and at these contact sites, there is evidence of calcium ion transfer from lysosomes to mitochondria, resulting in mitochondrial calcium overload and oxidative‐reductive imbalance. Reducing mitochondrial‐lysosomal contacts restores mitochondrial function and alleviates aging. Additionally, we observed that under conditions of induced aging, the proportion of peripheral division of mitochondria increases, which is mediated by mitochondrial‐lysosomal contacts. Moreover, under soft matrix stiffness, MSCs' lysosomes exhibit higher mobility and longer migration distances, suggesting that the rapid and disordered movement may increase the probability of lysosomal contact with mitochondria. We speculate that under aging conditions, lysosomes transfer calcium ions to mitochondria at the mitochondrial‐lysosomal contact sites, leading to mitochondrial calcium overload and oxidative‐reductive imbalance, with peripheral division of mitochondria potentially alleviating oxidative‐reductive stress, a process in which lysosomes are also involved. However, further research is needed to explore the specific mechanisms by which mitochondrial‐lysosomal contacts regulate crosstalk between mitochondria and lysosomes.

The extracellular microenvironment impacts the homeostasis of stem cells, with matrix stiffness playing a regulatory role in the aging and differentiation of these cells.^59^, ^60^ Previous studies have suggested that higher matrix stiffness in the extracellular matrix encourages osteogenic differentiation of MSCs, yet the association between matrix stiffness and stem cell aging remains inconclusive.^36^, ^37^ While some research has indicated that the stiffness of the bone marrow matrix increases with age, leading to aging of haematopoietic stem cells,^61^ other studies have proposed that MSCs cultured under higher matrix stiffness are less susceptible to aging, hinting at the tissue‐specific nature of determining matrix stiffness for aging.^62^, ^63^ For this reason, we evaluated the extracellular matrix stiffness of the microenvironment where MSCs were injected to treat cranial defects in mice. At the site of cranial defects in young mice, the extracellular matrix stiffness was approximately 30 kPa. Subsequent in vitro experiments reaffirmed that MSCs cultured under higher matrix stiffness were less prone to aging and displayed a higher likelihood of osteogenesis. Cells interpret extracellular matrix stiffness through diverse mechanisms, such as mechanosensitive ion channels, focal adhesions, cell–cell interactions, and interactions between the cell nucleus and cytoskeleton. Alterations in the cytoskeleton critically influence organelle behaviour, and it has been validated that lysosomes move along the cell's cytoskeleton. We confirmed that under softer matrix stiffness, the arrangement of the actin and tubulin cytoskeleton is more disordered, potentially leading to increased random movement of lysosomes, thereby heightening their likelihood of contacting mitochondria. Also, lysosomes are pivotal in regulating oxidative stress and may transfer their accumulated substances through organelle contacts under hydrogen peroxide induction.

## 5 CONCLUSION

In the process of osteogenesis in aged mice, the reduction in extracellular matrix stiffness leads to disordered rearrangement of the cellular cytoskeleton. In the aging environment, highly active lysosomes induce rapid movement along the disordered cytoskeleton, facilitating their easy recognition and contact with mitochondria. By increasing the number of mitochondria‐lysosome contacts, the peripheral fission of mitochondria, and the accumulation of punctate dysfunctional mitochondria, mitochondrial dysfunction is induced, subsequently causing intracellular homeostasis imbalance, increased accumulation of intracellular ROS, ultimately resulting in the aging and diminished osteogenic capacity of stem cells.

## AUTHOR CONTRIBUTIONS

*Conceptualization*: X.Z., K.W., C.H., and X.L. *Methodology*: K.W., C.H., X.L., and J.H. *Investigation*: K.W., C.H., and X.L. *Formal analysis*: K.W., C.H., X.L., and J.H. *Writing*: Q.L., J.W., Y.Y., and K.W. *Funding acquisition*: X.Z. *Supervision*: X.Z., K.W., C.H., and X.L.

## CONFLICT OF INTEREST STATEMENT

All authors disclosed no relevant relationships.

## Supporting information


**Data S1:** Supporting Information.

## Data Availability

The data that support the findings of this study are available from the corresponding author upon reasonable request.

## References

[1] Cooper C , Campion G , Melton LJ 3rd. Hip fractures in the elderly: a world‐wide projection. Osteoporosis Int. 1992;2(6):285‐289. 10.1007/BF01623184 1421796

[2] Johnell O , Kanis JA . An estimate of the worldwide prevalence and disability associated with osteoporotic fractures. Osteoporosis Int. 2006;17(12):1726‐1733. 10.1007/s00198-006-0172-4 16983459

[3] Kusumbe AP , Ramasamy SK , Adams RH . Coupling of angiogenesis and osteogenesis by a specific vessel subtype in bone. Nature. 2014;507(7492):323‐328. 24646994 10.1038/nature13145 PMC4943525

[4] Xie H , Cui Z , Wang L , et al. PDGF‐BB secreted by preosteoclasts induces angiogenesis during coupling with osteogenesis. Nat Med. 2014;20(11):1270‐1278. 25282358 10.1038/nm.3668 PMC4224644

[5] Zhou S , Greenberger JS , Epperly MW , et al. Age‐related intrinsic changes in human bone‐marrow‐derived mesenchymal stem cells and their differentiation to osteoblasts. Aging Cell. 2008;7(3):335‐343. 18248663 10.1111/j.1474-9726.2008.00377.x PMC2398731

[6] Lacolley P , Regnault V , Segers P , Laurent S . Vascular smooth muscle cells and arterial stiffening: relevance in development, aging, and disease. Physiol Rev. 2017;97(4):1555‐1617. 28954852 10.1152/physrev.00003.2017

[7] Rahmati M , Nalesso G , Mobasheri A , Mozafari M . Aging and osteoarthritis: central role of the extracellular matrix. Ageing Res Rev. 2017;40:20‐30. 28774716 10.1016/j.arr.2017.07.004

[8] Zhao Y , Simon M , Seluanov A , Gorbunova V . DNA damage and repair in age‐related inflammation. Nat Rev Immunol. 2023;23(2):75‐89. 35831609 10.1038/s41577-022-00751-y PMC10106081

[9] He S , Sharpless NE . Senescence in health and disease. Cell. 2017;169(6):1000‐1011. 28575665 10.1016/j.cell.2017.05.015 PMC5643029

[10] Xiao H , Jedrychowski MP , Schweppe DK , et al. A quantitative tissue‐specific landscape of protein redox regulation during aging. Cell. 2020;180(5):968‐983.e24. 32109415 10.1016/j.cell.2020.02.012 PMC8164166

[11] Zhang C , Li H , Li J , Hu J , Yang K , Tao L . Oxidative stress: a common pathological state in a high‐risk population for osteoporosis. Biomed Pharmacother. 2023;163:114834. 37163779 10.1016/j.biopha.2023.114834

[12] López‐Otín C , Blasco MA , Partridge L , Serrano M , Kroemer G . Hallmarks of aging: An expanding universe. Cell. 2023;186(2):243‐278. 36599349 10.1016/j.cell.2022.11.001

[13] Brunet A , Goodell MA , Rando TA . Ageing and rejuvenation of tissue stem cells and their niches. Nat Rev Mol Cell Biol. 2023;24(1):45‐62. 35859206 10.1038/s41580-022-00510-w PMC9879573

[14] Weng Z , Wang Y , Ouchi T , et al. Mesenchymal stem/stromal cell senescence: hallmarks, mechanisms, and combating strategies. Stem Cells Transl Med. 2022;11(4):356‐371. 35485439 10.1093/stcltm/szac004 PMC9052415

[15] Saraswathibhatla A , Indana D , Chaudhuri O . Cell‐extracellular matrix mechanotransduction in 3D. Nat Rev Mol Cell Biol. 2023;24(7):495‐516. 36849594 10.1038/s41580-023-00583-1 PMC10656994

[16] Shi H , Zhou K , Wang M , et al. Integrating physicomechanical and biological strategies for BTE: biomaterials‐induced osteogenic differentiation of MSCs. Theranostics. 2023;13(10):3245‐3275. 37351163 10.7150/thno.84759 PMC10283054

[17] Higuchi A , Ling QD , Chang Y , Hsu ST , Umezawa A . Physical cues of biomaterials guide stem cell differentiation fate. Chem Rev. 2013;113(5):3297‐3328. 23391258 10.1021/cr300426x

[18] Engler AJ , Sen S , Sweeney HL , Discher DE . Matrix elasticity directs stem cell lineage specification. Cell. 2006;126(4):677‐689. 16923388 10.1016/j.cell.2006.06.044

[19] Rajan TS , Scionti D , Diomede F , et al. Prolonged expansion induces spontaneous neural progenitor differentiation from human gingiva‐derived mesenchymal stem cells. Cell Reprogram. 2017;19(6):389‐401. 29058474 10.1089/cell.2017.0012

[20] Diomede F , Rajan TS , D'Aurora M , et al. Stemness characteristics of periodontal ligament stem cells from donors and multiple sclerosis patients: a comparative study. Stem Cells Int. 2017;2017:1606125. 29387088 10.1155/2017/1606125 PMC5745749

[21] Kauppila TES , Kauppila JHK , Larsson N‐G . Mammalian mitochondria and aging: an update. Cell Metab. 2017;25(1):57‐71. 28094012 10.1016/j.cmet.2016.09.017

[22] Smith KA , Waypa GB , Schumacker PT . Redox signaling during hypoxia in mammalian cells. Redox Biol. 2017;13:228‐234. 28595160 10.1016/j.redox.2017.05.020 PMC5460738

[23] Nunnari J , Suomalainen A . Mitochondria: in sickness and in health. Cell. 2012;148(6):1145‐1159. 22424226 10.1016/j.cell.2012.02.035 PMC5381524

[24] Kashatus DF , Lim KH , Brady DC , Pershing NLK , Cox AD , Counter CM . RALA and RALBP1 regulate mitochondrial fission at mitosis. Nat Cell Biol. 2011;13(9):1108‐1115. 21822277 10.1038/ncb2310 PMC3167028

[25] Yu SB , Pekkurnaz G . Mechanisms orchestrating mitochondrial dynamics for energy homeostasis. J Mol Biol. 2018;430(21):3922‐3941. 30089235 10.1016/j.jmb.2018.07.027 PMC6186503

[26] Chan DC . Mitochondrial dynamics and its involvement in disease. Annu Rev Pathol. 2020;15:235‐259. 31585519 10.1146/annurev-pathmechdis-012419-032711

[27] Wong YC , Ysselstein D , Krainc D . Mitochondria‐lysosome contacts regulate mitochondrial fission via RAB7 GTP hydrolysis. Nature. 2018;554(7692):382‐386. 29364868 10.1038/nature25486 PMC6209448

[28] Wong YC , Kim S , Peng W , Krainc D . Regulation and function of mitochondria‐lysosome membrane contact sites in cellular homeostasis. Trends Cell Biol. 2019;29(6):500‐513. 30898429 10.1016/j.tcb.2019.02.004 PMC8475646

[29] Kleele T , Rey T , Winter J , et al. Distinct fission signatures predict mitochondrial degradation or biogenesis. Nature. 2021;593(7859):435‐439. 33953403 10.1038/s41586-021-03510-6

[30] Ferrucci L , Fabbri E . Inflammageing: chronic inflammation in ageing, cardiovascular disease, and frailty. Nat Rev Cardiol. 2018;15(9):505‐522. 30065258 10.1038/s41569-018-0064-2 PMC6146930

[31] Cowan CM , Shi YY , Aalami OO , et al. Adipose‐derived adult stromal cells heal critical‐size mouse calvarial defects. Nat Biotechnol. 2004;22(5):560‐567. 15077117 10.1038/nbt958

[32] Yao S , Pang M , Wang Y , et al. Mesenchymal stem cell attenuates spinal cord injury by inhibiting mitochondrial quality control‐associated neuronal ferroptosis. Redox Biol. 2023;67:102871. 37699320 10.1016/j.redox.2023.102871 PMC10506061

[33] Gao Y , Chi Y , Chen Y , et al. Multi‐omics analysis of human mesenchymal stem cells shows cell aging that alters immunomodulatory activity through the downregulation of PD‐L1. Nat Commun. 2023;14(1):4373. 37474525 10.1038/s41467-023-39958-5 PMC10359415

[34] Huang X , Fan J , Li L , et al. Fast, long‐term, super‐resolution imaging with Hessian structured illumination microscopy. Nat Biotechnol. 2018;36(5):451‐459. 29644998 10.1038/nbt.4115

[35] Zhao W , Zhao S , Li L , et al. Sparse deconvolution improves the resolution of live‐cell super‐resolution fluorescence microscopy. Nat Biotechnol. 2022;40(4):606‐617. 34782739 10.1038/s41587-021-01092-2

[36] Hsieh WT , Liu YS , Lee YH , Rimando MG , Lin KH , Lee OK . Matrix dimensionality and stiffness cooperatively regulate osteogenesis of mesenchymal stromal cells. Acta Biomater. 2016;32:210‐222. 26790775 10.1016/j.actbio.2016.01.010

[37] Na J , Yang Z , Shi Q , et al. Extracellular matrix stiffness as an energy metabolism regulator drives osteogenic differentiation in mesenchymal stem cells. Bioact Mater. 2024;35:549‐563. 38434800 10.1016/j.bioactmat.2024.02.003 PMC10909577

[38] Qiu K , Zou W , Fang H , et al. Light‐activated mitochondrial fission through optogenetic control of mitochondria‐lysosome contacts. Nat Commun. 2022;13(1):4303. 35879298 10.1038/s41467-022-31970-5 PMC9314359

[39] Pu J , Guardia CM , Keren‐Kaplan T , Bonifacino JS . Mechanisms and functions of lysosome positioning. J Cell Sci. 2016;129(23):4329‐4339. 27799357 10.1242/jcs.196287 PMC5201012

[40] Zheng P , Obara CJ , Szczesna E , et al. ER proteins decipher the tubulin code to regulate organelle distribution. Nature. 2022;601(7891):132‐138. 34912111 10.1038/s41586-021-04204-9 PMC8732269

[41] Wang B , He W , Prosseda PP , et al. OCRL regulates lysosome positioning and mTORC1 activity through SSX2IP‐mediated microtubule anchoring. EMBO Rep. 2021;22(7):e52173. 33987909 10.15252/embr.202052173 PMC8406401

[42] Correia‐Melo C , Marques FD , Anderson R , et al. Mitochondria are required for pro‐ageing features of the senescent phenotype. EMBO J. 2016;35(7):724‐742. 26848154 10.15252/embj.201592862 PMC4818766

[43] Giorgi C , Marchi S , Pinton P . The machineries, regulation and cellular functions of mitochondrial calcium. Nat Rev Mol Cell Biol. 2018;19(11):713‐730. 30143745 10.1038/s41580-018-0052-8

[44] Sebastián D , Palacín M , Zorzano A . Mitochondrial dynamics: coupling mitochondrial fitness with healthy aging. Trends Mol Med. 2017;23(3):201‐215. 28188102 10.1016/j.molmed.2017.01.003

[45] Khacho M , Clark A , Svoboda DS , et al. Mitochondrial dynamics impacts stem cell identity and fate decisions by regulating a nuclear transcriptional program. Cell Stem Cell. 2016;19(2):232‐247. 27237737 10.1016/j.stem.2016.04.015

[46] Li Q , Gao Z , Chen Y , Guan MX . The role of mitochondria in osteogenic, adipogenic and chondrogenic differentiation of mesenchymal stem cells. Protein Cell. 2017;8(6):439‐445. 28271444 10.1007/s13238-017-0385-7 PMC5445026

[47] Li A , Gao M , Jiang W , Qin Y , Gong G . Mitochondrial dynamics in adult cardiomyocytes and heart diseases. Front Cell Dev Biol. 2020;8:584800. 33392184 10.3389/fcell.2020.584800 PMC7773778

[48] Zhang J , Nuebel E , Daley GQ , Koehler CM , Teitell MA . Metabolic regulation in pluripotent stem cells during reprogramming and self‐renewal. Cell Stem Cell. 2012;11(5):589‐595. 23122286 10.1016/j.stem.2012.10.005 PMC3492890

[49] Park JT , Lee YS , Cho KA , Park SC . Adjustment of the lysosomal‐mitochondrial axis for control of cellular senescence. Ageing Res Rev. 2018;47:176‐182. 30142381 10.1016/j.arr.2018.08.003

[50] Cui M , Yamano K , Yamamoto K , et al. HKDC1, a target of TFEB, is essential to maintain both mitochondrial and lysosomal homeostasis, preventing cellular senescence. Proc Natl Acad Sci USA. 2024;121(2):e2306454120. 38170752 10.1073/pnas.2306454120 PMC10786298

[51] Wang S , Long H , Hou L , et al. The mitophagy pathway and its implications in human diseases. Signal Transduct Target Ther. 2023;8(1):304. 37582956 10.1038/s41392-023-01503-7 PMC10427715

[52] König T , Nolte H , Aaltonen MJ , et al. MIROs and DRP1 drive mitochondrial‐derived vesicle biogenesis and promote quality control. Nat Cell Biol. 2021;23(12):1271‐1286. 34873283 10.1038/s41556-021-00798-4

[53] Jiménez‐Loygorri JI , Villarejo‐Zori B , Viedma‐Poyatos Á , et al. Mitophagy curtails cytosolic mtDNA‐dependent activation of cGAS/STING inflammation during aging. Nat Commun. 2024;15(1):830. 38280852 10.1038/s41467-024-45044-1 PMC10821893

[54] Guo Y , Guan T , Yu Q , et al. ALS‐linked SOD1 mutations impair mitochondrial‐derived vesicle formation and accelerate aging. Redox Biol. 2024;69:102972. 38056310 10.1016/j.redox.2023.102972 PMC10746562

[55] Hao T , Yu J , Wu Z , et al. Hypoxia‐reprogramed megamitochondrion contacts and engulfs lysosome to mediate mitochondrial self‐digestion. Nat Commun. 2023;14(1):4105. 37433770 10.1038/s41467-023-39811-9 PMC10336010

[56] Kim S , Wong YC , Gao F , Krainc D . Dysregulation of mitochondria‐lysosome contacts by GBA1 dysfunction in dopaminergic neuronal models of Parkinson's disease. Nat Commun. 2021;12(1):1807. 33753743 10.1038/s41467-021-22113-3 PMC7985376

[57] Peng W , Schröder LF , Song P , Wong YC , Krainc D . Parkin regulates amino acid homeostasis at mitochondria‐lysosome (M/L) contact sites in Parkinson's disease. Sci Adv. 2023;9(29):eadh3347. 37467322 10.1126/sciadv.adh3347 PMC10355824

[58] Peng W , Wong YC , Krainc D . Mitochondria‐lysosome contacts regulate mitochondrial Ca(2+) dynamics via lysosomal TRPML1. Proc Natl Acad Sci USA. 2020;117(32):19266‐19275. 32703809 10.1073/pnas.2003236117 PMC7430993

[59] Zheng C , Chen J , Liu S , Jin Y . Stem cell‐based bone and dental regeneration: a view of microenvironmental modulation. Int J Oral Sci. 2019;11(3):23. 31423011 10.1038/s41368-019-0060-3 PMC6802669

[60] Li Z , Yue M , Liu X , et al. The PCK2‐glycolysis axis assists three‐dimensional‐stiffness maintaining stem cell osteogenesis. Bioact Mater. 2022;18:492‐506. 35415308 10.1016/j.bioactmat.2022.03.036 PMC8971594

[61] Zhang X , Cao D , Xu L , et al. Harnessing matrix stiffness to engineer a bone marrow niche for hematopoietic stem cell rejuvenation. Cell Stem Cell. 2023;30(4):378‐395.e8. 37028404 10.1016/j.stem.2023.03.005

[62] Ogle ME , Doron G , Levy MJ , Temenoff JS . Hydrogel culture surface stiffness modulates mesenchymal stromal cell Secretome and alters senescence. Tissue Eng Part A. 2020;26(23–24):1259‐1271. 32628570 10.1089/ten.tea.2020.0030

[63] Šimoliūnas E , Ivanauskienė I , Bagdzevičiūtė L , Rinkūnaitė I , Alksnė M , Baltriukienė D . Surface stiffness depended gingival mesenchymal stem cell sensitivity to oxidative stress. Free Radic Biol Med. 2021;169:62‐73. 33862162 10.1016/j.freeradbiomed.2021.04.012

