# Supplementary material for: Matrix stiffness regulates mitochondria‐lysosome contacts to modulate the mitochondrial network, alleviate the senescence of MSCs
Source: Cell Prolif. 2024 Oct 1;58(2):e13746. doi: 10.1111/cpr.13746 (PMC11839199; doi:10.1111/cpr.13746)
Supplement: Supplementary file 1 — Data S1: Supporting Information. [file CPR-58-e13746-s001.docx]

**Matrix stiffness regulates mitochondria-lysosome contacts to modulate the mitochondrial network, alleviate the senescence of MSCs**


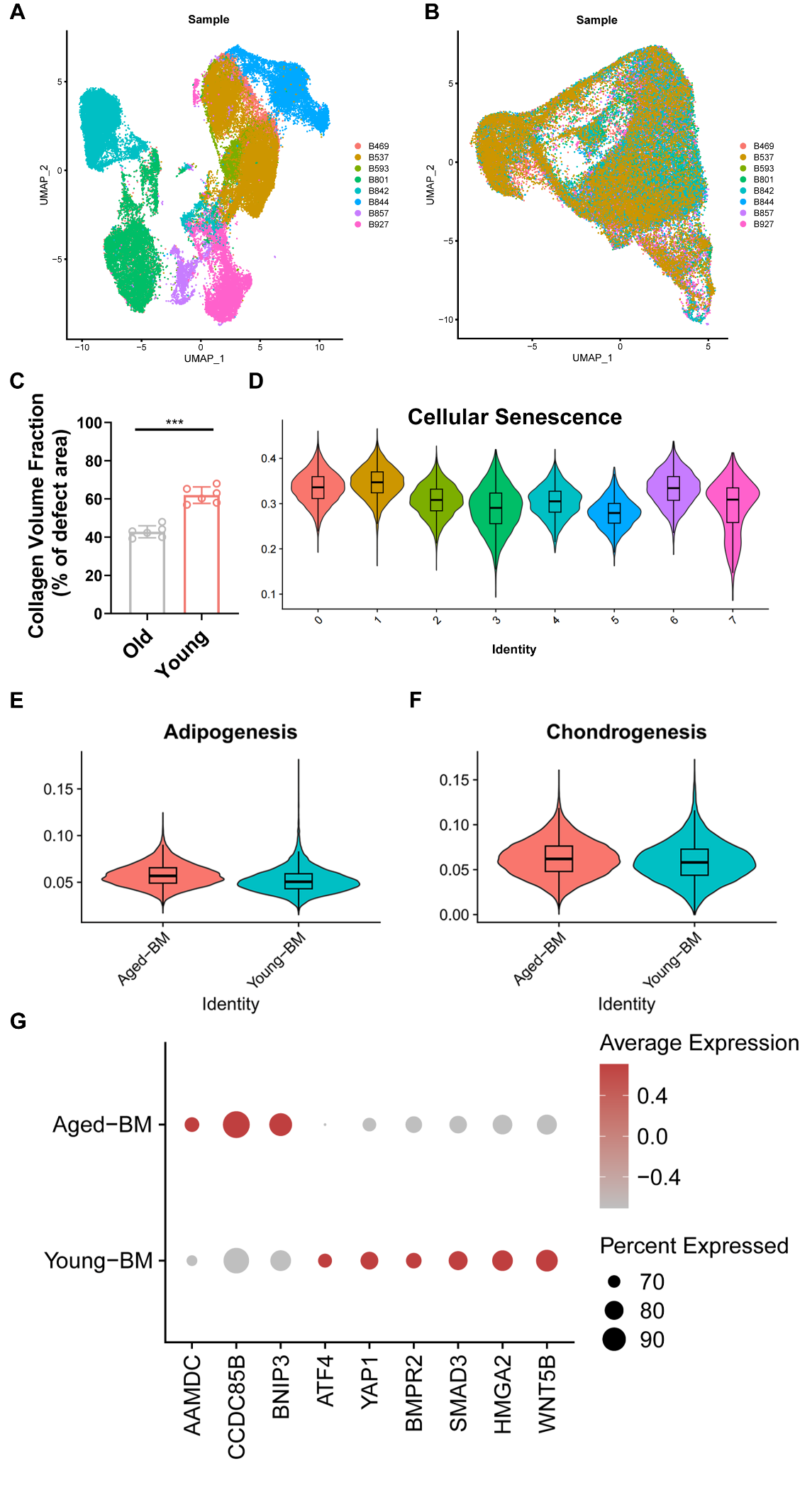


**Fig.S1.** **Assessment of senescence scoring and differentiation gene expression in MSCs subpopulations.**

**(A)**UMAP plots of the sample distribution before effect correction**. (B)**UMAP plots of the sample

distribution after effect correction**. (C)** The statistical graph represents the collagen volume fraction of the defect area at the fourth week. (**D)** Violin plots showing average expression of cellular senescence genes for each cluster. **(E-F)** Violin plots of the adipogenesis score, chondrogenesis score of young-BM and Aged-BM cluster. Box plot within each violin plot indicate median values, and the 25th to 75th percentiles. **(G)** Dot plots showing the expression values of representative trilineage differentiation-related genes for young-BM and Aged-BM cluster. Color represents the scaled expression values from Seurat RNA assay.


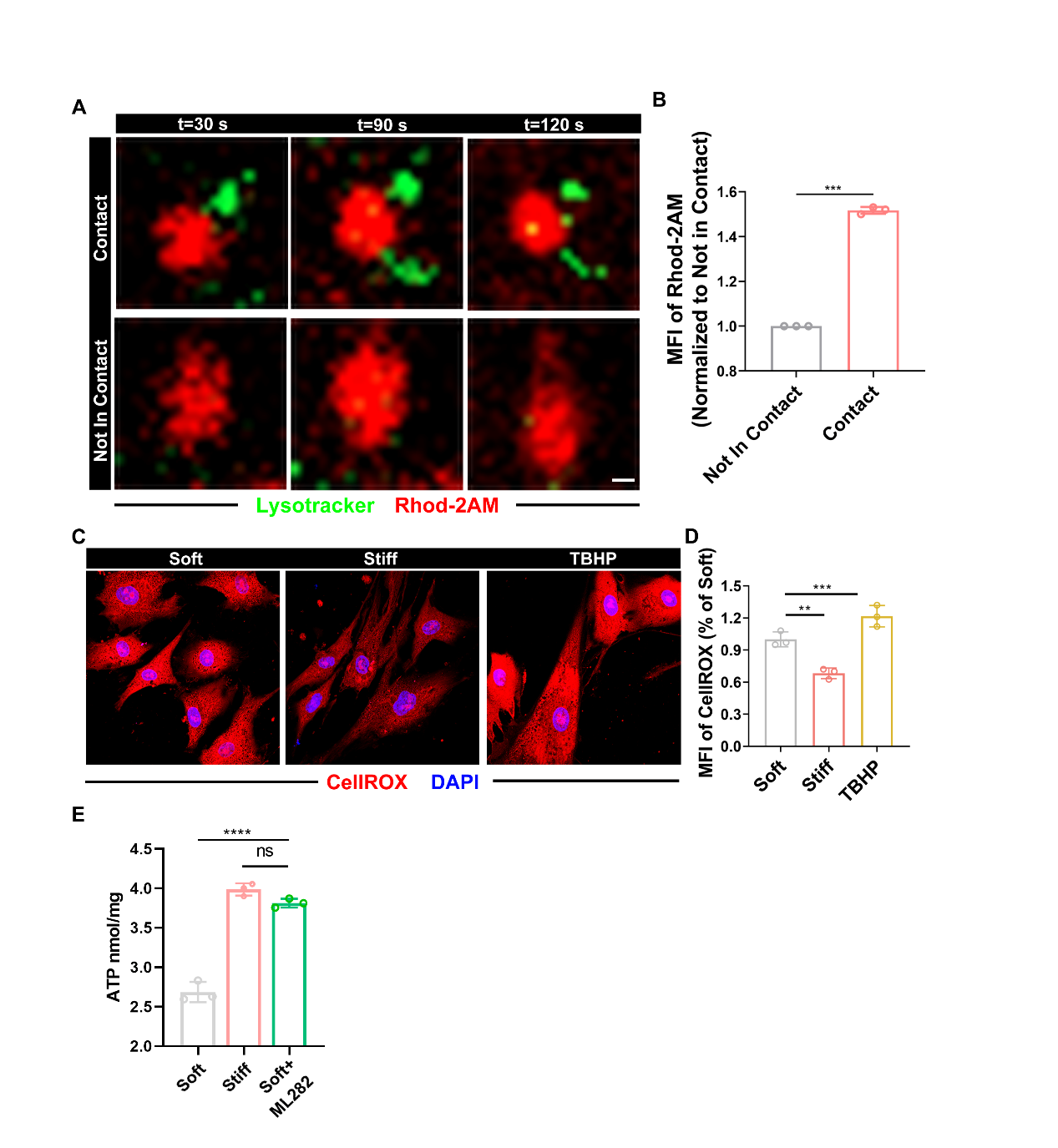


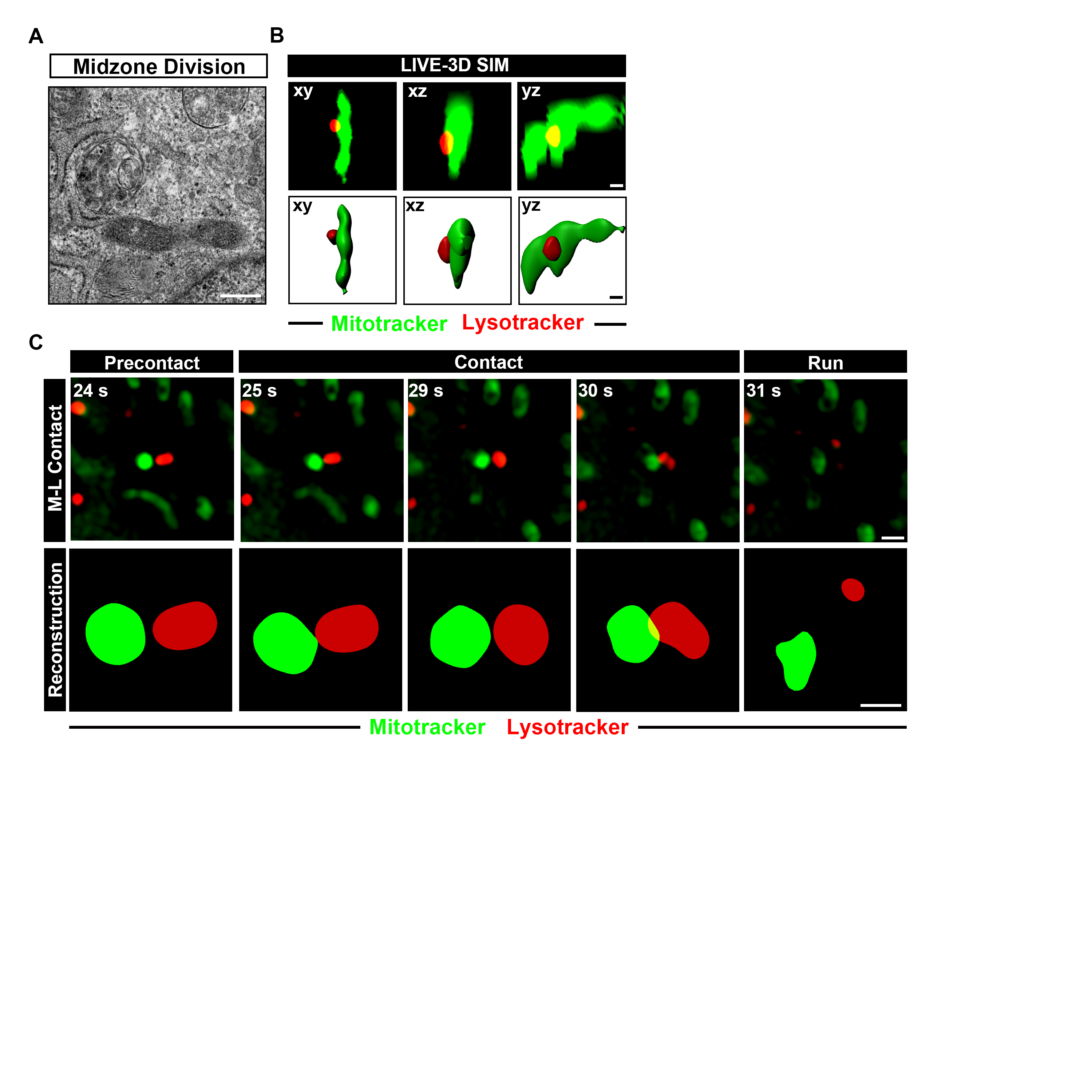


**Fig.S2.** **Three-dimensional reconstruction and dynamic imaging of mitochondria-lysosome contact.**

**(A)**Representative TEM images of the mitochondrial midzone division of MSCs on polyacrylamide gels induced by H_2_O_2_ at 48hr. Red arrows indicate contact area respectively. Scale bar, 500 nm **(B)** LIVE-3D SIM reconstruction images of mitochondria-lysosome contact. **(C)** Representative super-resolution living cell tracing images and reconstruction images of M-L contacts in MSCs on polyacrylamide gels induced by H_2_O_2_ at 48hr at different time points. Mitochondria were stained with Mitotracker green and Lysosome were stained with Lysotracker red. Scale bar, 12 μm for original pictures and 1 μm for enlarged pictures.


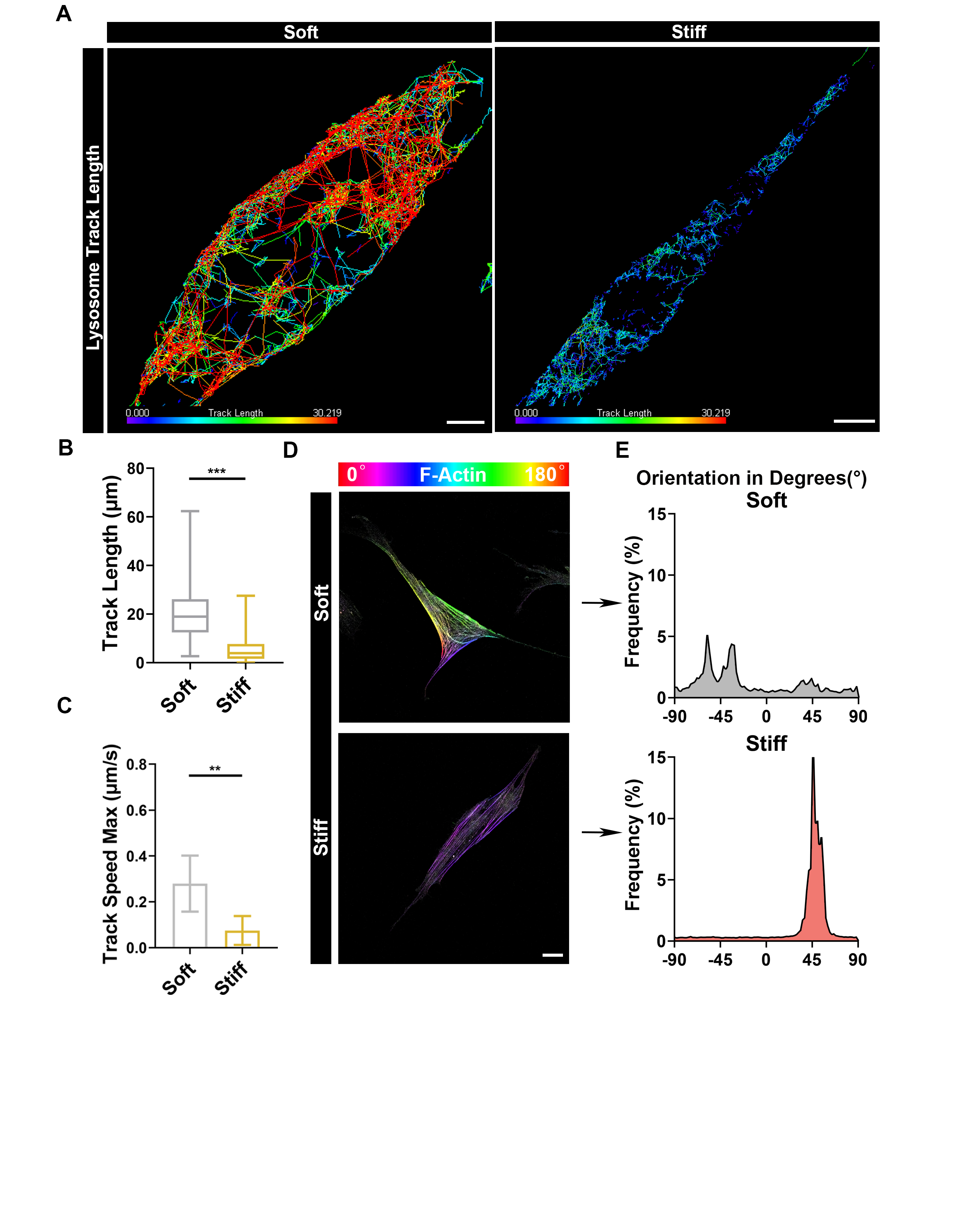


**Fig.S3.** **Lysosomal dynamics and cytoskeleton.**

**(A)** The representative super-resolution living cell tracing image of lysosome shows the track length. The color scale represents the length of the lysosome movement. The scale bar for the original image is 10 μm. (**B)** Quantitative statistical graph of the track length of figure A .

**(C)** Quantitative statistical graph of the track speed max of figure A . (**D)** Corresponding orientation plots for actin fiber staining, where the different colours indicate different orientations

of actin as per the given colourmap. Actin stress fibre microdomains can be identified by the uniformly coloured zones in the orientation plots. (**E)** Quantitative statistical graph for actin

staining orientations of figure D.


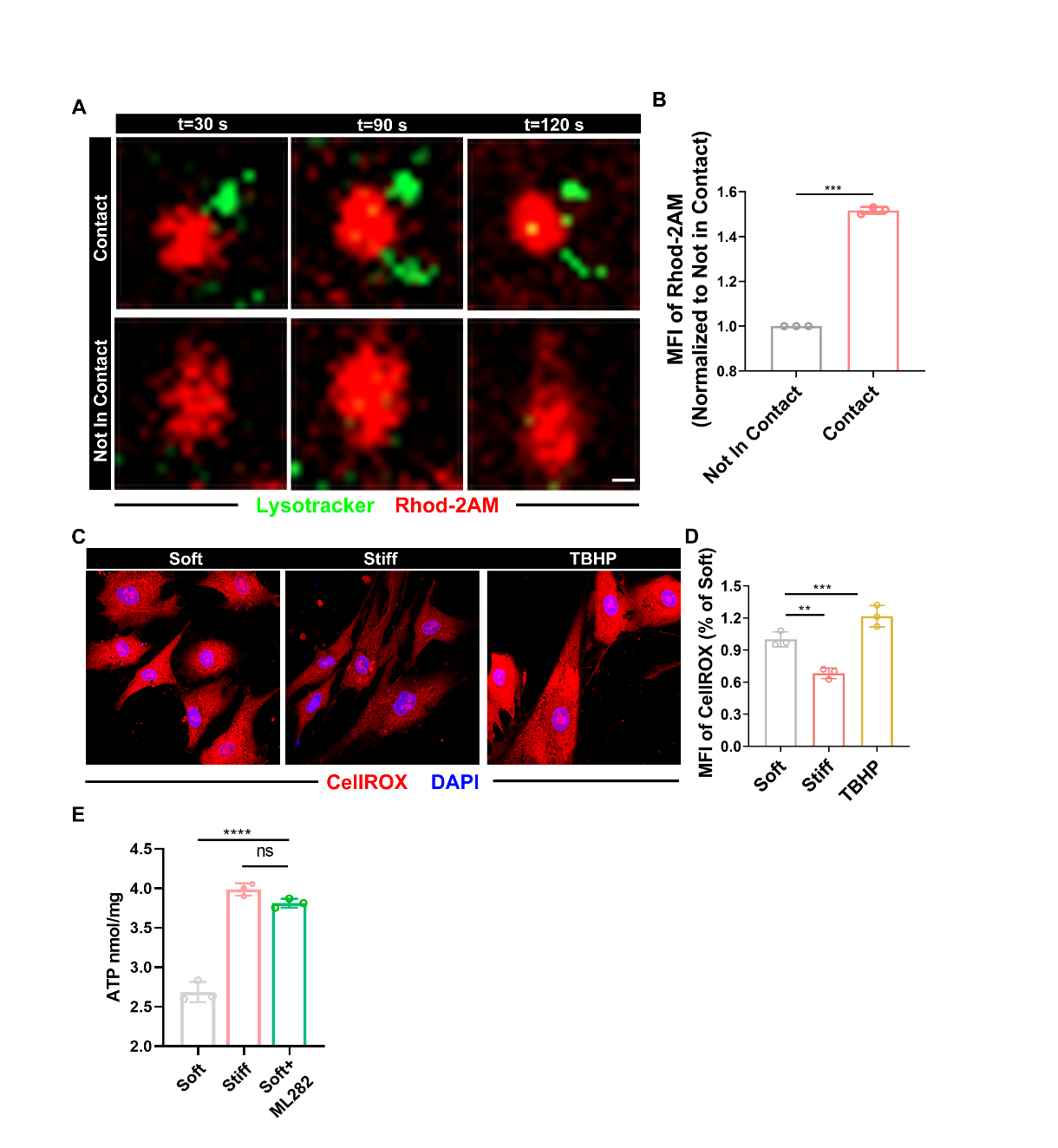


**Fig.S4.** **Calcium signaling at the mitochondria-lysosome interface and cellular ROS levels.**

**(A)** Representative living cell tracing images on confocal microscopy of the transport of calcium ions between M-L contacts in MSCs on polyacrylamide gels induced by H_2_O_2_ at 48hr at different time points. lysosomes were stained with Lysotracker green and calcium ion were stained with Rhod-2AM red. Scale bar, 500 nm. (**B)** Quantitative statistical analysis of median fluorescence intensity of calcium ion at M-L contact or non-contact in figure A.( **C)** Representative confocal microscopy images and quantitative analysis of the mean fluorescence intensity of of MSCs ROS(CellROX,red+) on soft and stiff olyacrylamide gels induced by H_2_O_2_ at 48hr. Scale bar,500 nm. TBHP: positive control. (**D)** Quantitative statistical analysis of median fluorescence intensity of Cell ROX in figure C. (**E)** The content of MSCs ATP on soft gels with ML282, soft and stiff olyacrylamide gels induced by H_2_O_2_ at 48hr.

**Supplementary table 1. Parameters used in processing of single-cell sequencing data**

| B469 | 3000 <nFeature_RNA< 7000 | percent.mt < 7.5 | 10000 <nCount_RNA< 55000 |
| --- | --- | --- | --- |
| B537 | 2250 <nFeature_RNA< 5750 | percent.mt < 6 | 5000 <nCount_RNA< 35000 |
| B593 | 4250 <nFeature_RNA< 7500 | percent.mt < 4.5 | 5000 <nCount_RNA< 55000 |
| B844 | 3250 <nFeature_RNA< 7000 | percent.mt < 7 | 10000 <nCount_RNA< 60000 |
| B801 | 3000 <nFeature_RNA< 6000 | percent.mt < 4.5 | 10000 <nCount_RNA< 45000 |
| B842 | 1750 <nFeature_RNA< 5750 | percent.mt < 5 | 5000 <nCount_RNA< 35000 |
| B857 | 1000 <nFeature_RNA< 10000 | percent.mt < 4 | 0 <nCount_RNA< 270000 |
| B927 | 3750 <nFeature_RNA< 8250 | percent.mt < 4 | 15000 <nCount_RNA< 110000 |

**Supplementary table 2.** **Functional scores gene list**

**Cellular Senescence (MsigDB: FRIDMAN_SENESCENCE_UP)**

| HSPA2 | CDKN2A | SERPINE1 | CDKN2B | CYP1B1 | CCND1 |
| --- | --- | --- | --- | --- | --- |
| RRAS | RHOB | FILIP1L | NRG1 | RAB31 | CCN2 |
| VIM | IGFBP4 | MMP1 | RAB5B | S100A11 | GUK1 |
| MAP2K3 | MAP1LC3B | CXCL14 | IRF5 | CITED2 | HTATIP2 |
| CDKN2D | IGFBP3 | NME2 | ISG15 | NDN | IGFBP2 |
| RBL2 | TSPYL5 | CLTB | IRF7 | IGFBP7 | F3 |
| IGFBP6 | TNFAIP3 | TP53 | IGFBP5 | HBS1L | ALDH1A3 |
| RAC1 | STAT1 | IFNG | IGSF3 | THBS1 | IFI16 |
| ING1 | CDKN1C | OPTN | RGL2 | CREG1 | SOD1 |
| CRYAB | COL1A2 | HPS5 | RABGGTA | SMURF2 | PEA15 |
| AOPEP | RAB13 | MDM2 | TFAP2A | TGFB1I1 | SPARC |
| TNFAIP2 | SERPINB2 | TES | CD44 | IGFBP1 | FN1 |
| CDKN1A | EIF2S2 | ESM1 | SMPD1 | GSN |  |

**Adipogenesis (GO:0045444 FAT_CELL_DIFFERENTIATION)**

| HDAC6 | ARL4A | CDS1 | CEBPA | CEBPB | CEBPD |
| --- | --- | --- | --- | --- | --- |
| SH2B2 | PPARGC1A | ADIRF | FERMT2 | CCDC85B | WIF1 |
| DUSP10 | OSBPL11 | C1QTNF3 | LRG1 | JDP2 | TTC8 |
| CMKLR1 | TAF8 | ATF2 | CREBL2 | MAPK14 | TMEM120B |
| ADIG | ADRB1 | ADRB2 | ADRB3 | ZFPM1 | DDIT3 |
| BBS12 | TMEM64 | DIO2 | E2F1 | EGR2 | FLCN |
| ZBTB7C | AKT1 | AKT2 | FABP4 | FABP3 | ADGRF5 |
| VSTM2A | NAPEPLD | FGF10 | TRIM32 | FOXO1 | PLCB1 |
| SIRT1 | ZFPM2 | ALOX5 | FRZB | FNDC5 | NOCT |
| ZNF385A | DHRS7B | CLIP3 | GATA2 | GATA3 | BSCL2 |
| GDF10 | ADGRF1 | DKKL1 | BBS9 | GNB3 | NUDT7 |
| METRNL | PTGR3 | GPER1 | FFAR2 | GRK5 | GPX1 |
| TRIB2 | AAMDC | ERO1A | SOX8 | NR4A1 | HES1 |
| HTR2A | HTR2C | FFAR4 | C1QL4 | IL6 | IL11 |
| INHBB | INS | PTPRQ | LAMB3 | GDF6 | LEP |
| LPL | LRP5 | BMAL1 | MIR103A1 | MIR106A | MIR107 |
| MIR17 | MIR181A2 | MIR21 | MIR27A | MIR27B | MIR29B1 |
| SMAD6 | MB | MMP11 | ALDH6A1 | MSX2 | NPR2 |
| NR4A2 | ZBTB7B | PRLH | SDF4 | PLAC8 | MEX3C |
| SIRT6 | PDGFRA | ENPP1 | ERAP1 | PIM1 | RETREG1 |
| PPARD | PPARG | TRPM4 | PID1 | ASXL2 | MIR448 |
| LMO3 | ANGPTL8 | SLC7A10 | RETN | PSMB8 | PTGS2 |
| TRIB3 | BBS1 | BBS2 | BBS4 | RARRES2 | TRPV4 |
| ALOXE3 | CCND1 | RGS2 | RNASEL | RORA | RORC |
| MIR483 | SORT1 | PRDM16 | SFRP1 | SFRP2 | NOC3L |
| EBF2 | BMP2 | SLC2A4 | BMP7 | SNAI2 | DLK2 |
| BNIP3 | SOD2 | SREBF1 | ZFP36L1 | ZFP36L2 | SULT1E1 |
| STK3 | STK4 | KLF5 | CNTN2 | MIR548D1 | TFAP2B |
| TGFB1 | TGFB1I1 | TNF | TPH1 | TRIO | UCP1 |
| WNT1 | WNT10B | XBP1 | ZFP36 | ZBTB16 | FTO |
| STEAP4 | ATAT1 | NR4A3 | ZC3H12A | PNPLA3 | HMGA2 |
| AXIN1 | AXIN2 | CCDC3 | TLCD3B | TMEM120A | ARID5B |
| LRRC8C | FAM120B | MEDAG | PIAS1 | RUNX1T1 | SOCS1 |
| PEX11A | CCN4 | PER2 | WNT3A | KLF4 | ADIPOQ |
| NR1D1 | GDF3 | SOX13 | ZNF516 | MAFB |  |

**Osteogenesis (GO:0001649 OSTEOBLAST_DIFFERENTIATION)**

| RUNX2 | BGLAP | MIR675 | MIR665 | TOB1 | ALYREF |
| --- | --- | --- | --- | --- | --- |
| CDK6 | LRRC17 | TCIRG1 | YAP1 | SYNCRIP | CEBPA |
| SEMA4D | MYBBP1A | TWIST2 | CLIC1 | CLTC | SP7 |
| DNAI3 | MAPK14 | CTNNB1 | CYP24A1 | DDX5 | DHX9 |
| TMEM64 | DLX5 | JAG1 | EPHA2 | RANBP3L | AKT1 |
| FBL | ESRRA | FASN | FBN2 | UCMA | SCUBE3 |
| FGF9 | FGFR2 | RRAS2 | SNRNP200 | ZHX3 | SATB2 |
| DNAJC13 | UFL1 | GTPBP4 | CLEC5A | ALPL | GABBR1 |
| NPNT | BAMBI | NOCT | ASF1A | RSL1D1 | FBXO5 |
| AMELX | GDF2 | GREM1 | GDF10 | SND1 | RBMX |
| GLI1 | GLI2 | GLI3 | GSK3B | SOX8 | HGF |
| HNRNPC | HNRNPU | HOXA2 | HSD17B4 | HSPE1 | TNC |
| IARS1 | IBSP | FFAR4 | TMEM119 | RSPO2 | OSTN |
| IGF1 | IGF2 | CCN1 | IHH | IL6R | ILK |
| AREG | LRP3 | LRP5 | LTF | MIR100 | MIR106A |
| MIR125B1 | MIR138-1 | MIR140 | MIR17 | MIR20A | MIR200C |
| MIR205 | MIR208A | MIR21 | MIR210 | MIR214 | MIR27A |
| MIR29B1 | MIR9-1 | MIR93 | MIR98 | SMAD1 | SMAD3 |
| SMAD4 | SMAD5 | SMAD6 | MEF2C | MEF2D | MEN1 |
| ACHE | MIR346 | CITED1 | MSX2 | MYOC | ATF4 |
| NELL1 | NF1 | NOTCH1 | NPPC | DDR2 | ATP5F1B |
| DHH | LEF1 | CRIM1 | SUCO | SIRT7 | HDAC7 |
| SUFU | WWOX | PHB1 | WNT4 | PPARG | TRPM4 |
| GDPD2 | PPP3CA | HEMGN | LGR4 | TENT5A | PRKACA |
| BCAP29 | MAPK11 | MAP2K6 | FAM20C | CTNNBIP1 | TWSG1 |
| PTCH1 | MIR20B | PTHLH | PTH1R | PTK2 | IFT80 |
| RDH14 | TP53INP2 | REST | RORB | CCL3 | TNN |
| FIGNL1 | SMOC1 | SFRP1 | SFRP2 | SHH | SHOX2 |
| SKI | BMP2 | BMP3 | BMP4 | BMP6 | BMP7 |
| BMPR1A | BMPR1B | BMPR2 | SNAI2 | SMO | SNAI1 |
| SOX2 | SOX9 | SOX11 | SPP1 | MIR548D1 | TNF |
| TNFAIP6 | HIRA | TWIST1 | WNT3 | WNT7B | WNT10B |
| WNT11 | TMEM53 | RIOX1 | FGF23 | AXIN2 | FZD1 |
| CAT | SEMA7A | TP63 | CHRD | CBFB | CCN4 |
| WNT3A | LIMD1 | ACVR1 | CREB3L1 | DDX21 | ACVR2A |
| NOG | ACVR2B | HAND2 | RASSF2 | NR1I3 |  |

**Chondrogenesis (GO:0002062 CHONDROCYTE_DIFFERENTIATION)**

| SIX2 | ARID5A | ADAMTS7 | OSR2 | MBOAT2 | COL11A1 |
| --- | --- | --- | --- | --- | --- |
| OSR1 | COMP | ATF2 | MAPK14 | RFLNA | CCN2 |
| CTNNB1 | CHADL | ECM1 | EXT1 | EXT2 | FGF9 |
| FGFR3 | CHSY1 | SLC39A14 | TSKU | AMELX | GLI3 |
| GPLD1 | ANXA6 | HOXA11 | IHH | RFLNB | HES5 |
| MUSTN1 | GDF6 | SNX19 | LTBP3 | SMAD3 | SMAD7 |
| MAF | MATN1 | MDK | MEF2C | MEF2D | MSX2 |
| NFIB | CCN3 | NPPC | NPR2 | CHST11 | ZNF219 |
| MEX3C | CYTL1 | POR | BPNT2 | SMPD3 | SOX6 |
| PRKG2 | SULF2 | TWSG1 | PTH | PTHLH | PTH1R |
| IFT80 | PTPN11 | NKX3-2 | RARB | RARG | RB1 |
| SFRP2 | SCX | SHOX2 | CREB3L2 | BMP2 | BMP4 |
| BMP6 | BMPR1A | BMPR1B | BMPR2 | SNAI2 | SOX5 |
| SOX9 | TGFB1 | TGFBR1 | TGFBR2 | TRPS1 | WNT7A |
| WNT10B | WNT2B | WNT9A | ZBTB16 | LNPK | HMGA2 |
| WNT5B | ADAMTS12 | GDF5 | AXIN2 | COL27A1 | SCIN |
| RUNX2 | SERPINH1 | FGF18 | CCN4 | PKDCC | ACVRL1 |
| EIF2AK3 |  |  |  |  |  |

**Supplementary Table 3.** Primers name and sequence.

| Primer name | Primer sequence |
| --- | --- |
| H-P16-F1 | 5'-GATCCAGGTGGGTAGAAGGTC-3' |
| H-P16-R1 | 5'-CCCCTGCAAACTTCGTCCT-3' |
| H-P21-F1 | 5'-TGTCCGTCAGAACCCATGC-3' |
| H-P21-R1 | 5'-AAAGTCGAAGTTCCATCGCTC-3' |
| H-P53-F1 | 5'-CAGCACATGACGGAGGTTGT-3' |
| H-P53-R1 | 5'-TCATCCAAATACTCCACACGC-3' |
| H-GAPDH-F1 | 5'-GGAGCGAGATCCCTCCAAAAT-3' |
| H-GAPDH-R1 | 5'-GGCTGTTGTCATACTTCTCATGG-3' |
| H-Runx2-F1 | 5'-TGGTTACTGTCATGGCGGGTA-3' |
| H-Runx2-R1 | 5'-TCTCAGATCGTTGAACCTTGCTA-3' |
| H-ALP-F1 | 5'-GTGAACCGCAACTGGTACTC-3' |
| H-ALP-R1 | 5'-GTGAACCGCAACTGGTACTC-3' |
| H-OCN-F1 | 5'-GTGCAGAGTCCAGCAAAGGT-3' |
| H-OCN-R1 | 5'-TCAGCCAACTCGTCACAGTC-3' |
| H-PPARy-F1 | 5'-GGGATCAGCTCCGTGGATCT-3' |
| H-PPARy-R1 | 5'-TGCACTTTGGTACTCTTGAAGTT-3' |
| H-Adipoq-F1 | 5'-GACACCAAAAGGGCTCAGGAT-3' |
| H-Adipoq-R1 | 5'-GAGTGCCATCTCTGCCATCA-3' |
| H-LPL-F1 | 5'-TTGAGTATGCAGAAGCCCCG-3' |
| H-LPL-R1 | 5'-GCTGGTCCACATCTCCAAGT-3' |
| H-FABP4-F1 | 5'-ACTGGGCCAGGAATTTGACG-3' |
| H-FABP4-R1 | 5'-CTCGTGGAAGTGACGCCTT-3' |
| H-NFE2L2-F | 5'-TACTCCCAGGTTGCCCACA-3' |
| H-NFE2L2-R | 5'-TACTCCCAGGTTGCCCACA-3' |
| H-HMOX1-F | 5'-ATTTCAGAAGGGCCAGGTGA-3' |
| H-HMOX1-R | 5'-GGAAGTA GACAGGGGCGAAGA-3' |
| H-NQO1-F | 5'-AAAGGACCCTTCCGGAGTAA-3' |
| H-NQO1-R | 5'-AGGCTGCTTGGAGCAAAATA-3' |
| H-GCLM-F | 5'-AGACGGGGAACCTGCTGAA-3' |
| H-GCLM-R | 5'-CATCTGGAAACTCCCTGACCA-3' |
| H-LAMP1-R | 5'-CACCACCCTCCTGTTGCTGTA-3' |
| H-GAA-F | 5'-CATCCTACTCCATGATTTCCTGC-3' |
| H-GAA-R | 5'-AGCTGGGTGAGTCCTCC-3' |
| H-CTSD-F | 5'-TGCTCAAGAACTACATGGACGC-3' |
| H-CTSD-R | 5'-CGAAGACGACTGTGAAGCACT-3' |
| H-CTSF-F | 5'-AAAGTCAAAGACCAGGGCA-3' |
| H-CTSF-R | 5'-TTGTCACAGTCCAAGAGCT-3' |
